# Bibliometric analysis of deep learning for surgical instrument segmentation, detection and tracking in minimally invasive surgery

**DOI:** 10.3389/fdgth.2026.1633888

**Published:** 2026-02-27

**Authors:** Mahmoud Yousef, Kareem Essam Aly, Mariam Ahmed, Fatimaelzahraa Ali Ahmed, Khalid Al Jalham, Shidin Balakrishnan

**Affiliations:** 1 Weill Cornell Medicine Qatar, Doha, Qatar; 2 College of Medicine, Qatar University, Doha, Qatar; 3 Department of Surgery, Hamad Medical Corporation, Doha, Qatar

**Keywords:** bibliometric analysis, minimally invasive surgery, surgical instrument detection and tracking, surgical instrument segmentation, surgical video analysis

## Abstract

**Background:**

Deep learning (DL) methods for surgical video analysis have expanded rapidly in minimally invasive surgery (MIS). However, a structured bibliometric overview focused on DL-based surgical instrument segmentation, detection, and tracking is lacking. The objective of this review is to systematically map the research landscape with this focus, by examining publication trends, influential authors, institutions, and countries, collaboration networks, keyword co-occurrence patterns, and the thematic trajectory of the discipline.

**Methods:**

We performed a bibliometric analysis of original research articles on DL-based surgical instrument segmentation/detection/tracking in laparoscopic or robotic MIS, published between 2017 and 2024. Searches were conducted in six databases namely PubMed, Scopus, IEEE Xplore, Embase, Medline, and Web of Science. Records were de-duplicated in EndNote and analyzed using the Bibliometrix R package, with co-authorship, co-citation, and keyword networks visualized in VOSviewer. Citation counts were extracted from each study's respective database and interpreted cautiously given the influence of publication age.

**Results:**

We included 217 articles. Annual output increased from 2017 to a peak in 2023, indicating sustained growth in DL research for MIS instrument analysis. The most productive countries included the United States and France, with major institutional contributions from the University of Strasbourg and Furtwangen University. Keyword analysis indicated continued dominance of convolutional neural networks alongside emerging themes including transformer-based architectures, multimodal learning, and real-time intraoperative applications.

**Conclusions:**

This bibliometric study characterizes the evolution, leading contributors, collaboration patterns, and thematic trajectories of DL-based instrument segmentation/detection/tracking in MIS. While these findings can inform research prioritization and collaboration, this study does not evaluate clinical effectiveness. Future work should prioritize explainable and efficient real-time models, standardized annotation protocols, and broader global partnerships to support responsible clinical translation.

## 1 Introduction

Minimally invasive surgery (MIS) represents a broad class of operative techniques that reduce surgical tissue damage by utilizing advanced intraoperative visualization. MIS includes laparoscopy and robotic-assisted surgery. In MIS, an endoscope is inserted into the patient's body through a small incision to give a clear and magnified view of the surgical site. Robotic-assisted surgery allows surgeons to control instruments from a console without direct interaction with the patient. The number of laparoscopic surgeries performed each year has exceeded 15 million (1) and robotic surgeries have represented over 15% of cases in fields like general surgery (2) and over 85% for certain procedures such as prostatectomies (3). Since these procedures use cameras, surgeries are often recorded. This has led to the development of publicly available surgical datasets, including benchmark resources from the MICCAI Endoscopic Vision (EndoVIS) challenges and datasets like HeiChole, that have helped facilitate advancements in the field (4, 5).

Computer vision and machine learning, subsets of Artificial Intelligence (AI), have played an important role in turning raw surgical video footage into processed and annotated datasets that can then be used for research and in clinical settings. Recently, advancements in deep learning (DL) techniques has enabled substantial improvements in modelling MIS, especially coupled with the growth of surgical data volume. Computer vision tasks like detecting objects, identifying instruments, segmenting crucial anatomical areas and recognition of surgical phase in surgical videos rely heavily on DL models. The exceptional performance of DL in various applications suggests active potential for automation in surgery, aiming to provide real-time insights during operations and supports surgeons in making better decisions. Furthermore, the development and release of open-source foundation models have been transformative, enabling researchers to collaborate and implement DL into surgical practices rapidly. For example, DL has been applied in instrument segmentation, surgical phase recognition, or anatomical landmark detection. For example, models like EndoNet (6) and SV-RCNet (7), as well as learning-based approaches for surgical phase recognition based on temporal modeling of instrument usage and video context (8) have all contributed significantly in advancing real-time workflow recognition and tool classification in laparoscopic videos. Other promising innovations include the use of Vision Transformers (ViTs) (9, 10) for surgical tool detection or Generative Adversarial Networks (GANs) (11, 12) for enhancing video quality.

These technical advancements suggest a growing importance of DL in MIS, yet the literature has not been systematically analyzed. These gaps leave significant questions unanswered regarding the intellectual structure of the field, the collaborative networks driving its growth, and the emerging trends that may shape its future. Similar bibliometric approaches have been used in other specialized fields to elucidate research impact and thematic evolution (13). By providing a structured, data-driven overview of impact and collaboration patterns, such analyses play a critical role in guiding future research priorities, identifying opportunities for meaningful collaboration, and supporting the coordinated advancement of the field in a coherent and informed manner.

This study aims to address the aforementioned gap by conducting a detailed bibliometric analysis of current research on DL in MIS, gathered from six major databases. It analyzes the most cited studies in the field, specifically those using deep learning for instrument segmentation, detection, and tracking in MIS. We included only papers that developed DL models due to their superior performance on complex tasks, such semantic segmentation, to: (1) examine publication trends and the growth trajectory of the field, (2) identify influential authors, institutions, and countries driving advancements in DL for MIS, (3) analyze collaboration networks and citation patterns to understand the field's intellectual and social structure, (4) explore keyword co-occurrence and emerging research topics, and (5) propose future research directions based on identified trends.

This bibliometric review provides a structured overview of the evolution of research on DL in MIS. By analyzing trends in publications, citations, collaboration patterns, and research topics, the study offers insight into the organization and progression of the field. Unlike clinical studies or meta-analyses that primarily assess technical performance or clinical accuracy, this analysis is intended to inform researchers about broader research patterns and gaps in the literature, offering a foundation for future work by supporting informed study design and collaboration.

## 2 Methodology

This bibliometric analysis was conducted in accordance with established methodological conventions used in previously published bibliometric studies across various clinical domains, ensuring methodological rigor (13–21). Accordingly, this study has been designed and reported in accordance with the BIBLIO checklist for bibliometric analyses (completed checklist provided as Supplementary File S1).

### 2.1 Search strategy

A comprehensive search was performed across six databases namely PubMed, Scopus, IEEE Xplore, Embase, Medline, and Web of Science using keywords like “robotic-assisted surgery,” “minimally invasive surgery,” “deep learning,” and “computer vision”, for the time period 2017–2024. We limited the analysis to 2017–2024 for two reasons. First, 2017 corresponds to the earliest year in which deep learning–based approaches for surgical instrument analysis in minimally invasive/video-guided surgery were consistently represented in major indexing services, coinciding with the emergence of widely used community benchmarks and challenges (e.g., EndoNet-era laparoscopic video recognition and the EndoVis challenge series (6). Second, 2024 was the most recent complete publication year at the time of our search. We did not include 2025 because the year was ongoing during data collection and indexing and citation accrual would be incomplete.

We included papers that focused on laparoscopic or robotic-assisted surgeries besides utilizing DL models such as Convolutional Neural Networks (CNNs), Long Short-Term Memory (LSTM) networks, Generative Adversarial Networks (GANs), or Vision Transformers (ViT). Because instrument-related surgical video studies are not consistently labeled with instrument-specific keywords in titles and abstracts, we intentionally used a high-sensitivity search strategy focused on MIS + deep learning + video/endoscopy terms. Specificity was then enforced during screening using predefined eligibility criteria requiring a direct link to instrument segmentation, detection, or tracking.

The search strategy is shown in Table 1 below:

**Table 1:** Search strategy.

Concept	Keywords and MeSH Terms
Minimally Invasive Surgery	Keywords: laparoscop* OR “partial nephrectomy” OR endoscop* OR adrenalectomy OR cholecystectomy OR splenectomy OR nephrectomy OR “general surgery” OR “minimally invasive surgical procedur*” OR “minimally invasive surgery” OR “surgical procedur*” OR “operation room” OR “ surgery robot*” OR “surgical robot*”
	MeSH term: “Minimally Invasive Surgical Procedures”[Mesh] “Surgical Procedures, Operative”[Mesh]
	Search: “Minimally Invasive Surgical Procedures”[Mesh] OR “Surgical Procedures, Operative”[Mesh] OR laparoscop* OR “partial nephrectomy” OR endoscop* OR adrenalectomy OR cholecystectomy OR splenectomy OR nephrectomy OR “general surgery” OR “minimally invasive surgical procedur*” OR “minimally invasive surgery” OR “surgical procedur*” OR “operation room” OR “ surgery robot*” OR “surgical robot*”
Deep Learning	Keywords:“artificial intelligence” OR “machine learning” OR “Image-guided Surgery” OR “deep learning” OR “artificial neural network*” OR “neural network*” OR “convolutional neural network*”
	MeSH terms: “Artificial Intelligence”[Mesh]
	Search: “Artificial Intelligence"[Mesh] OR “artificial intelligence” OR “machine learning” OR “Image-guided Surgery” OR “deep learning” OR “artificial neural network*” OR “neural network*” OR “convolutional neural network*"
Video	Keywords: video* OR “Video Recording*” OR “Video-Assisted Surgery” MeSH term: “Video Recording”[Mesh]
	Search: “Video Recording”[Mesh] OR video* OR “Video Recording*” OR “Video-Assisted Surgery”

### 2.2 Screening & inclusion/exclusion criteria

A thorough process was implemented to ensure the inclusion of eligible studies. Firstly, the search results were exported in RIS format and imported into Endnote 21.3 (Clarivate Analytics, Philadelphia, PA, USA). Duplicate records were then removed, after which all retrieved studies underwent independent title and abstract screening by two reviewers, followed by full-text assessment by two reviewers to determine eligibility. Discrepancies were resolved by consensus. This strategy ensured high sensitivity at the search stage while maintaining strict specificity through subsequent screening based on predefined eligibility criteria.

Our exclusion criteria included research focusing solely on conventional image processing or non-DL machine learning models. Additionally, studies that were applied to ex-vivo testing or simulation kits were not considered. Secondary research like reviews and meta-analyses were excluded to maintain the focus on original studies. Furthermore, studies focusing on workflow recognition without a direct connection to surgical tool detection were also excluded, though those incorporating tool detection as part of surgical phase identification were included.

### 2.3 Data extraction

Data were extracted clearly and systematically from the 217 included papers. The extracted metadata included the title, list of authors, year of publication, citation count, title of the journal, publisher, institutional affiliations of authors, and keywords. Citation counts were extracted from each study's respective indexing database. We report absolute citation counts as primary bibliometric indicators. Because citations accumulate over time and are influenced by publication age and indexing practices, we also report citations per year (total citations divided by years since publication) for the most-cited studies and interpret cross-country and cross-institution comparisons cautiously. Field-normalized metrics such as field-weighted citation impact (FWCI) were not used because they were not consistently available across all retrieved records. Papers with 50 citation counts or above were analyzed critically, by extracting the DL methodology, type of procedure and application. This approach helped us understand the correlation between the popularity of these papers and the direction of research. Later, the metadata of the 217 papers were analyzed using specific bibliometric software application. It allows the identification of important patterns of research, the identification of influential authors, and the study of relationships in the structure.

### 2.4 Bibliometric analysis techniques

After duplicates removal, the RIS file containing bibliometric data of the 217 studies was imported into Bibliometrix (22) a bibliometrics analysis tool driven by R programming language (version 4.1.3; R Foundation for Statistical Computing, Vienna, Austria). The data in this file was processed in a systematic manner to create tables and figures representing major trends and emergent patterns in the research landscape. The mapping of the co-authorship and citation patterns was created using the VOSviewer 1.6.20 (23) software package developed at Leiden University by Van Eck. Hence, these visualizations allowed deep insights into the relations among countries, authors, and institutions and thus shed light on the collaborative nature of research in DL in MIS.

## 3 Results

### 3.1 Growth of publications over time

**Figure 1:**
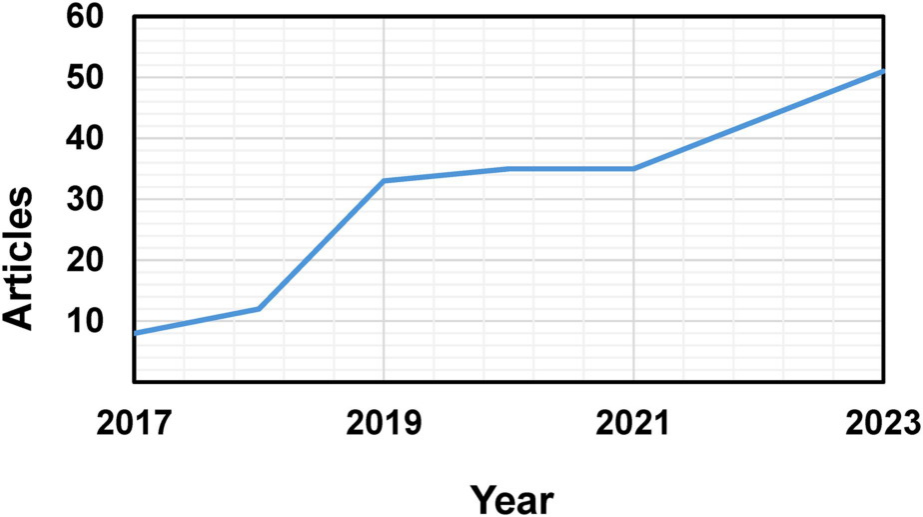
Total number of articles published per year, displaying the growth of publications in the field.

The utilization of DL in the segmentation of surgical instruments for MIS has seen substantial advancement over the last eight years (Figure 1). The initial articles on surgical data recognition via deep learning first appeared in 2017, starting with eight papers (6, 24–30). The sector experienced consistent progress, resulting in a linear growth trajectory start in 2021. During this interval, an average of 35 papers were published per year over two consecutive years, indicating that the publication rate had reached a plateau. Nonetheless, this plateau was momentary, as the field resumed its upward trend, ultimately reaching a peak in 2023 and exceeding 50 publications.

### 3.2 Distribution by publication type

Since 2017, 217 papers on the application of DL for surgical instrument recognition in MIS have been published, with an average citation around 20 per paper. These include 128 journal articles and 89 conference papers (Table 2). Different journals and conferences showed interest in this topic, resulting in 93 various sources. The average number of authors per article was 6.27, with 25.11% of them involving international co-authorships.

**Table 2:** Summary of relevant characteristics of publications including general information, document contents, authors, and document types.

Characteristic	Value
General information	
Timespan	2017–2024
Number of Various Sources (Journals, Conferences, etc.)	93
Number of Documents	217
Annual Growth Rate %	9.43
Average Years since Publication	3.06
Average citations per doc	19.7
References	6,179
Document contents	
Keywords Plus (ID)	1,501
Author's Keywords (DE)	482
Authors	
Authors	974
Authors of single-authored docs	5
Single-authored docs	5
Average Number of Co-Authors per Doc	6.27
International co-authorships (%)	25.11
Document types	
Articles	128
Conference Papers	89

### 3.3 Distribution of publications by country

**Figure 2:**
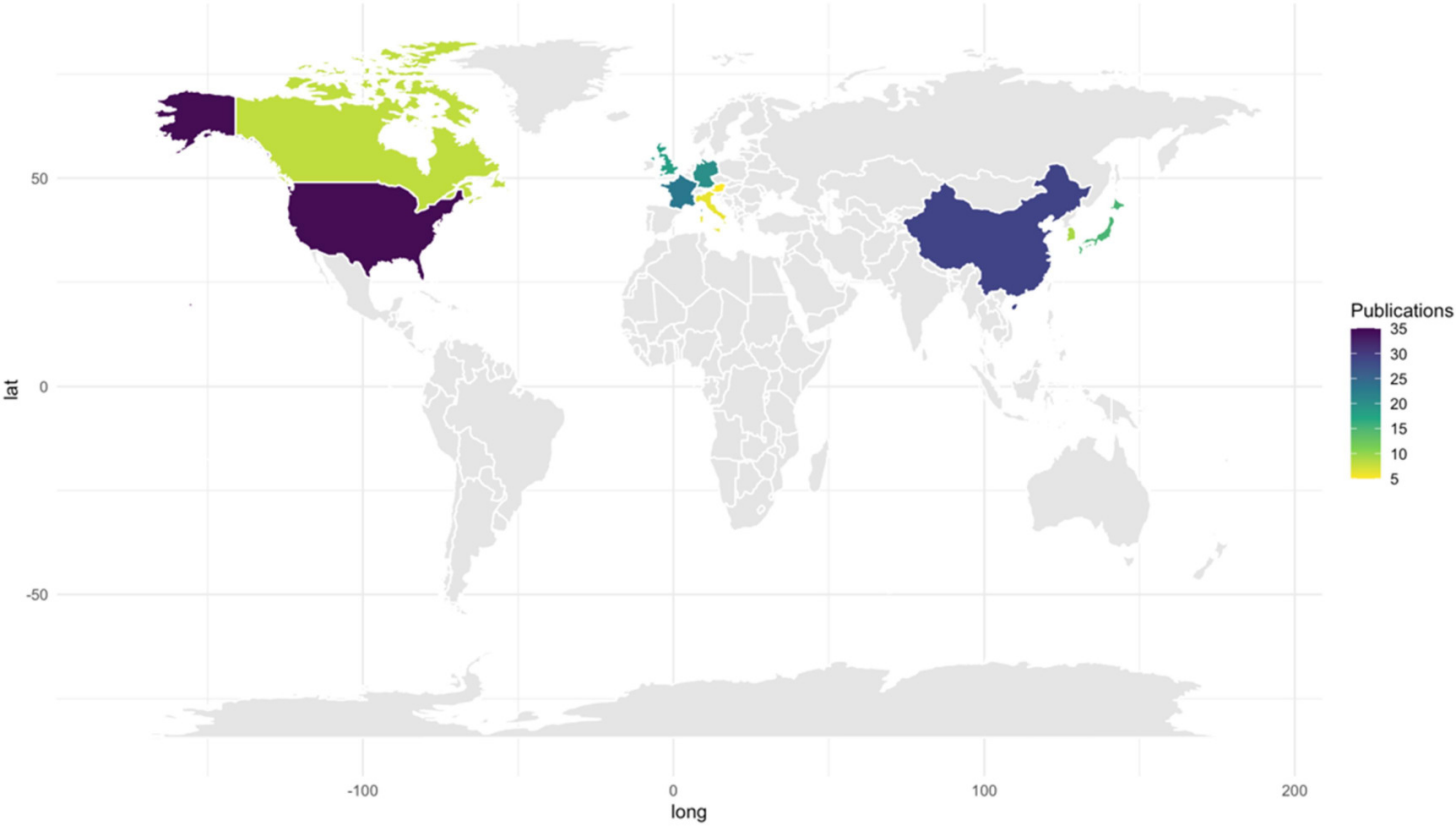
Number of publications by country. The country of publication is determined by the geographic location of the primary authors' institution.

Geographical analysis reveals that many countries have made significant contributions to the field (Figure 2). The United States leads in the total number of publications (35) (26, 31–64), followed by China (25) (44–68) and Germany (24) (24, 65–87) Notably, France also stands out with 19 publications (6, 29, 88–103), and the United Kingdom (104–121) and Japan (8, 102–117) make considerable contributions with 18 and 17 publications, respectively. The data indicates that a large portion of publications, totaling 88, originate from Europe, with substantially fewer contributions from regions such as Latin America and Africa. Countries in East Asia, including Hong Kong, South Korea, Japan, Vietnam, and China, represent a significant proportion of contributions from that region.

### 3.4 Distribution of publications by institution

**Figure 3:**
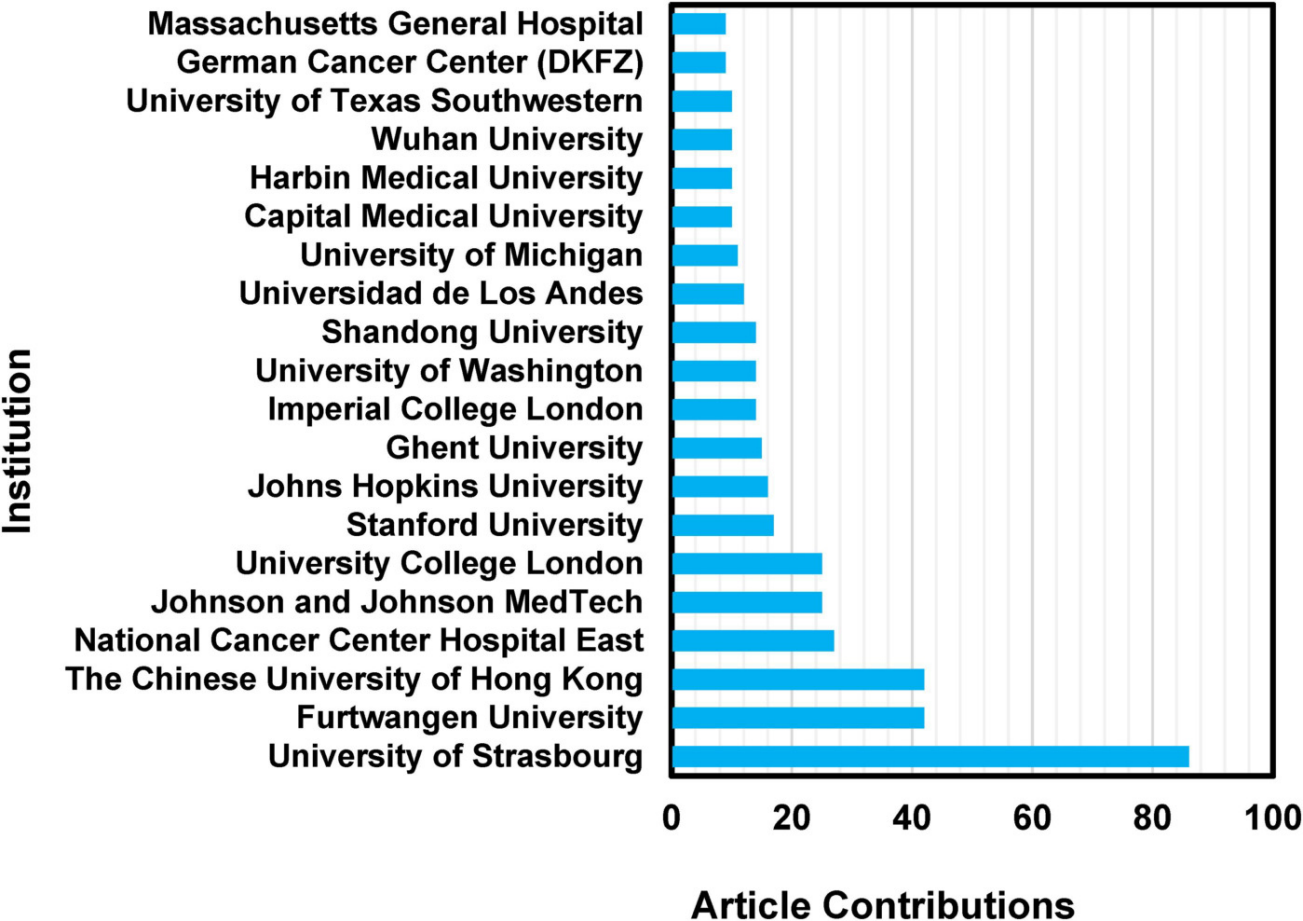
Number of article contributions by institution, regarding the top twenty institutions in the field. An article contribution for an institution is counted if at least one of the authors on the source belongs to that respective institution.

A total of 370 institutions contributed to at least one article relevant to the production of the 217 included papers (Figure 3). The distribution of contributing institutions spans several countries, with notable contributions from France, Germany, the United States, the United Kingdom, and China. France's contributions were primarily driven by institutions like the University of Strasbourg (86 articles). German institutions like Furtwangen University (42 articles) and the German Cancer Research Center (DKFZ) (9 articles) also featured prominently. In the United States, Stanford University (17 articles) and Johns Hopkins University (16 articles), among others, were notable contributors. Additionally, it is relevant to note that two institutions with 11 articles were not included in the figure, namely the University of Texas Southwestern Medical Center and the Shanghai Jiao Tong University.

### 3.5 Distribution of publications by author

A total of 974 authors have contributed to articles in this field, however, a few prominent authors emerge (Table 3). Padoy (6, 8, 53, 69, 73, 88–91, 93, 95, 96, 98, 99, 101, 103, 122) has the most at 17 publications from the University of Strasbourg, followed closely by Stoyanov (53, 91, 102, 106, 109, 110, 113, 114, 116–121, 123) from University College of London with 15. Next, Jalal (65, 66, 68, 70, 71, 74–77, 80, 82, 86, 117) from Furtwangen and Mutter (6, 88, 90, 91, 94, 96, 98, 99, 101, 103) from Strasbourg, have 13 and 10 publications, respectively. Heng (25, 63, 106, 124–129). from the Chinese University of Hong Kong contributed 9 publications, while Jin (106, 124, 126, 130–133), Moeller (65, 66, 68, 70, 71, 74, 76, 77), Mascagni, (53, 88, 91, 95, 96, 98, 99, 122), Neumuth (65, 68, 71, 74–78), and Nwoye (69, 88–91, 96, 99, 101) have 8 article contributions each.

**Table 3:** Number of publications for the ten most published authors.

Author	Publications
Padoy N.	17
Stoyanov D.	15
Jalal N.A.	13
Mutter D.	10
Heng P.A.	9
Jin Y.	8
Mascagni P.	8
Moeller K.	8
Neumuth T.	8
Nwoye C.	8

### 3.6 Distribution of citations by country

**Figure 4:**
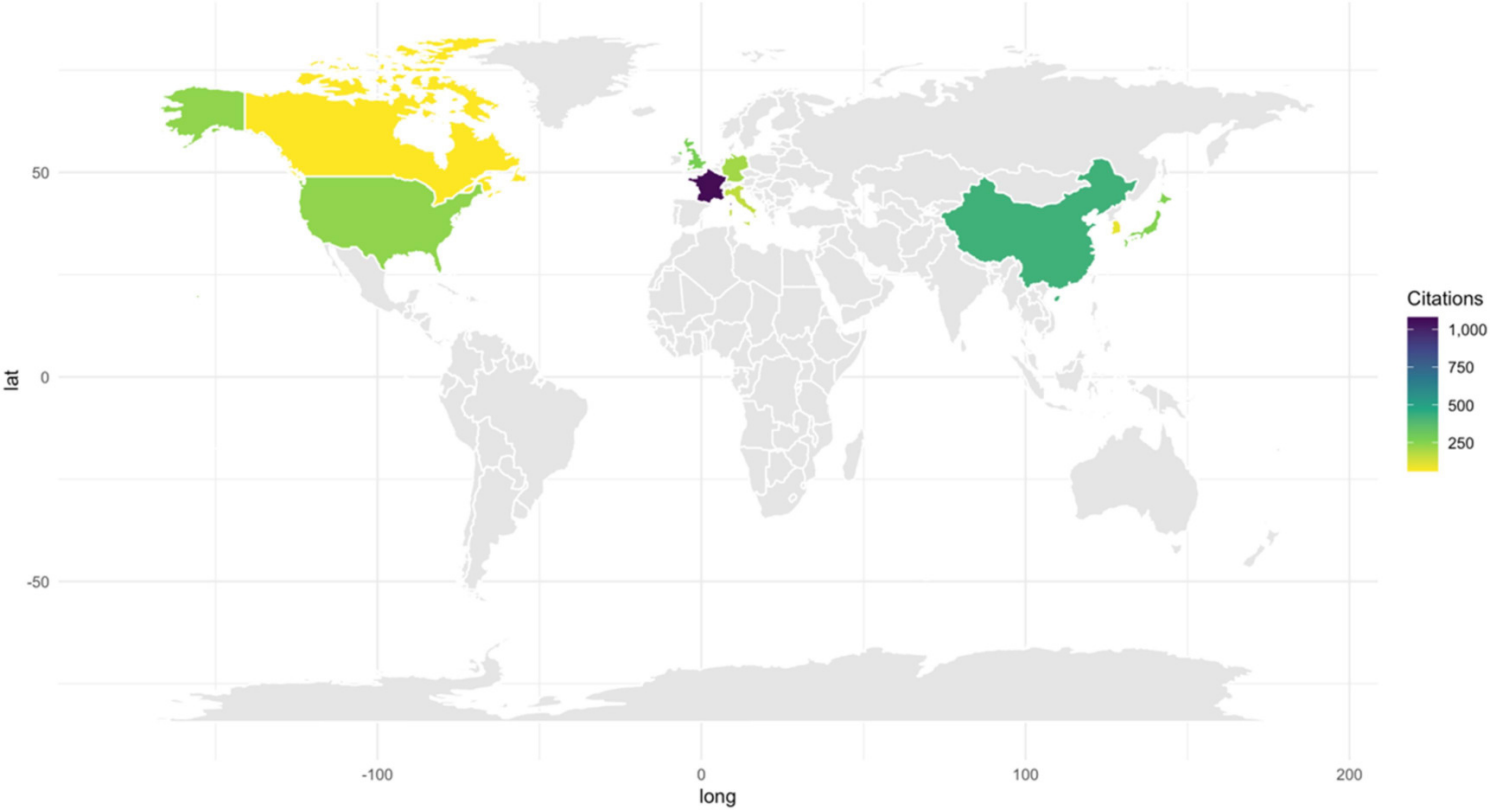
Number of citations by country. The country of citation is determined by the geographic location of the primary authors' institution.

The analysis of the published studies showed that the countries with the highest number of citations were also the countries with the largest number of contributions (Figure 4). France has more than 1,000 citations, making it the country with the highest citation count, followed by Hong Kong (424). United Kingdom and China have the same number of citations at 273, followed by Japan at 254. Most of the top 10 most cited countries have over 100 citations, however, South Korea (97) and Canada (60) fall below this.

### 3.7 Most frequently used keywords

The top five most occurring keywords were “Laparoscopy” (223), “Deep Learning” (206), “Convolutional Neural Network” (115), “Surgical Equipment” (101), and “Robotic Surgery” (96) (Table 4). Further, “Videorecording” (86) and “Endoscopic Surgery” (62) were frequently mentioned.

**Table 4:** Number of keyword occurrences in the included publications, showing the top ten.

Keyword	Occurrences
Laparoscopy	223
Deep Learning	206
Convolutional Neural Network	115
Surgical Equipment	101
Robotic Surgery	96
Videorecording	86
Endoscopic Surgery	62
Cataract Surgery	61
Transplant Surgery	52
Workflow	48

### 3.8 Emerging trends in keywords over time

**Figure 5:**
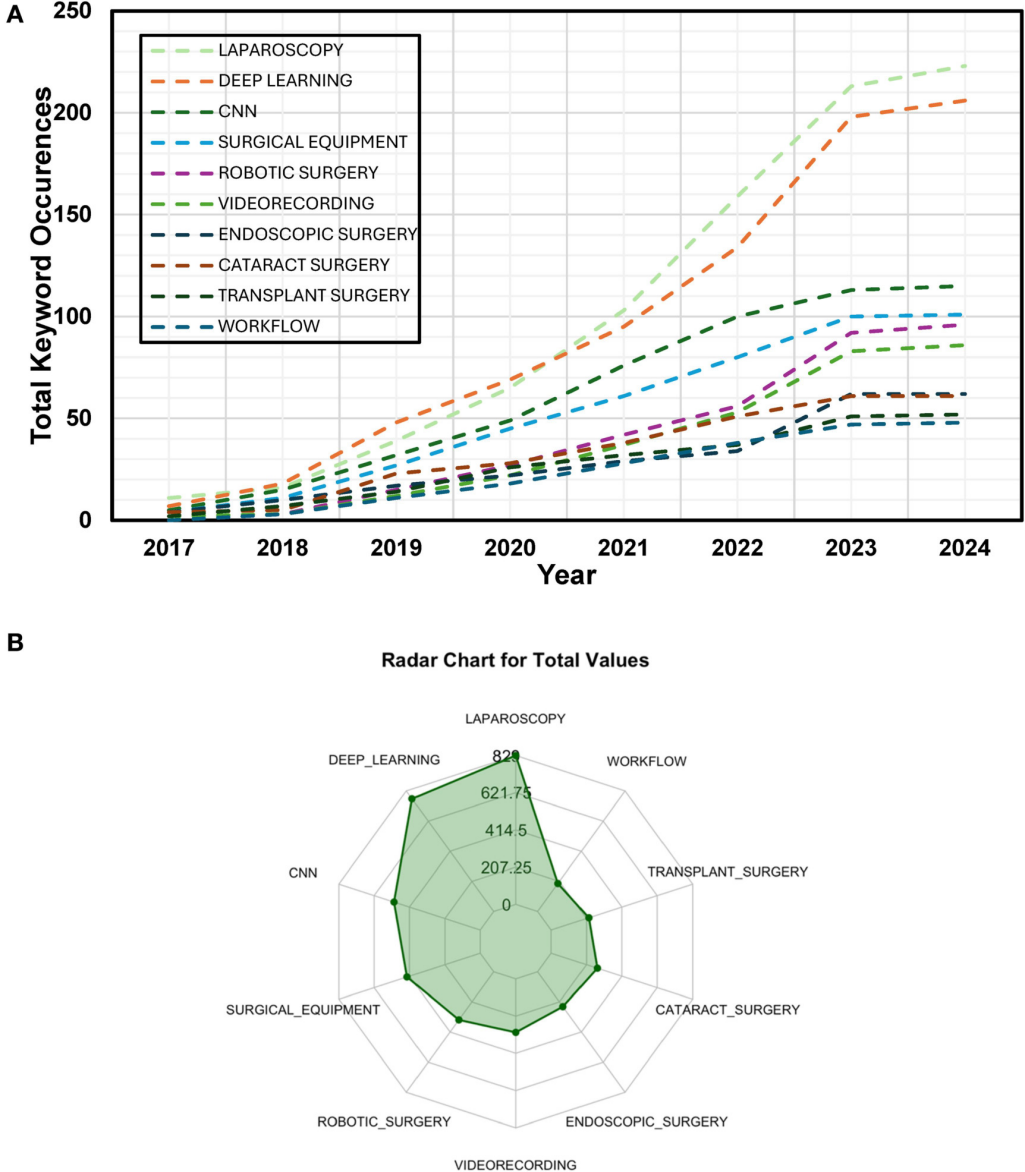
**(A)** Total number of keyword occurrences in the included publications from 2017 to 2024. **(B)** Total number of keyword occurrences in the included publications from 2017 to 2024.

The timeline (Figure 5A) clearly shows a growing trend in research activity of the keywords, especially from 2017 onwards, which is consistent with the number of article publications displayed in Figure 1. For example, mentions of “deep learning” increased from just 3 occurrences in 2017 to 74 in 2021, reaching 206 by the end of 2023. Similarly, “laparoscopy” grew significantly during the same period. Figure 5B illustrates the total occurrences of the top keywords. Although “Laparoscopy” dominates in frequency, other specific surgeries categorized under it, such as “Endoscopic Surgery,” “Transplant Surgery,” and “Cataract Surgery,” are also frequently found.

### 3.9 Distribution of publications by journal

Although there were 93 sources in the form of journals and conference proceedings, a few specific journals stood out in terms of total number of publications. The journal with the highest number of articles published was the International Journal of Computer Assisted Radiology and Surgery (24, 36, 38, 41, 54, 56, 67, 73, 78, 83, 90, 98, 101, 109, 110, 117, 119, 126, 134–144), with 27 articles and an impact factor (IF) of 2.3. Lecture Notes in Computer Science (25, 58, 69, 84, 99, 103, 104, 118, 121, 130, 145–153) came next with 19 articles published followed by Medical Image Analysis (88, 89, 91, 93, 96, 102, 113, 124, 127, 154, 155) with a high IF of 10.9. Surgical Endoscopy (31, 40, 42, 50, 53, 95, 156–159) and IEEE Transactions on Medical Imaging (6, 7, 32, 34, 106, 114, 120, 125, 160, 161) both had 10 publications; however, they had IFs of 2.4 and 10.6, respectively. The next 5 journals all had fewer than 10 articles in each and IFs of less than 5.

### 3.10 Top cited papers

The ten most cited articles using DL for surgical tool detection in MIS is shown in Table 5. Most of the highly cited articles are published by IEEE, with a few contributions published by Elsevier, JAMA and Springer. The most cited paper is “EndoNet: A Deep Architecture for Recognition Tasks on Laparoscopic Videos,” (6) authored by Twinanda (2017, 924 citations) which is published in IEEE. The paper proposed a CNN model performs surgical tools classification and workflow recognition. This paper is one of the first papers in the field of instruments segmentation using DL model. Shvets' “Automatic Instrument Segmentation in Robot-Assisted Surgery using Deep Learning” (60) (2018, 433 citations), which explored binary, semantic and tools’ parts segmentation using CNN blocks in an encoder-decoder fashion. The model was more specific compared to other models as it was developed for robotic-assisted surgeries. Jin's 2018 article, “Tool Detection and Operative Skill Assessment in Surgical Videos Using Region-Based Convolutional Neural Networks” (61), with 327 citations, utilizes instrument segmentation to assess the surgeons' skill level for training purposes.

**Table 5:** The ten most cited articles in the field, alongside their year of publication, primary author, total citations, and publisher.

Title	Year	Primary author	Total citations	Publisher	Application	Dl algorithms
EndoNet: A Deep Architecture for Recognition Tasks on Laparoscopic Videos (6)	(2017)	Twinada	924	IEEE	Tools classifications and workflow recognition	CNNs
Automatic Instrument Segmentation in Robot-Assisted Surgery using Deep Learning (60)	(2018)	Shvets	433	IEEE	Binary, semantic, tools’ parts segmentation	CNNs with encoder decoder architecture
Tool Detection and Operative Skill Assessment in Surgical Videos Using Region-Based Convolutional Neural Networks (61)	(2018)	Jin	327	IEEE	Multiclass classification and detection using bounding box	CNNs
SV-RCNet: Workflow Recognition From Surgical Videos Using Recurrent Convolutional Network (7)	(2018)	Jin	274	IEEE	Classification of instruments and workflow recognition	Recurrent convolution neural networks
Multi-task recurrent convolutional network with correlation loss for surgical video analysis (127)	(2020)	Jin	179	El Selvier ScienceDirect	Workflow recognition and instrument classification	Recurrent convolutional network, spatio-temporal features, very deep residual network, long short-term memory.
Machine and deep learning for workflow recognition during surgery (162)	(2019)	Padoy	148	Taylor and Francis	Surgical workflow recognition and tool detection	CNNs and LSTM
Weakly supervised convolutional LSTM approach for tool tracking in laparoscopic videos (101)	(2019)	Nwoye	144	Springer Link	Real-time binary surgical tool tracking	CNN + Convolutional LSTM (ConvLSTM)
Articulated Multi-Instrument 2-D Pose Estimation Using Fully Convolutional Networks (120)	(2018)	Du	140	IEEE	Instruments joints detection	FCN
Deep Learning Based Robotic Tool Detection and Articulation Estimation With Spatio-Temporal Layers (163)	(2019)	Colleoni	115	IEEE	Surgical instrument joint detection and localization	Three dimensional CNN
Assessment of Automated Identification of Phases in Videos of Cataract Surgery Using Machine Learning and Deep Learning Techniques (164)	(2019)	Yu	106	JAMA	Instrument classification and workflow recognition	CNN-RNN

The number of citations were verified using Google Scholar.

Moreover, Du's “Articulated Multi-Instrument 2-D Pose Estimation Using Fully Convolutional Networks” (120) (2018, 40 citations), focuses on pose estimation in laparoscopic surgery, relying on segmentation for accurate tool positioning. The accurate positioning was implemented through the detection of instruments joints. Colleoni has also developed a three-dimensional CNN that detects and localize surgical tools joints, published under “Deep Learning Based Robotic Tool Detection and Articulation Estimation With Spatio-Temporal Layers” (2019, 115 citations). Jin's (163) “Multi-task recurrent convolutional network with correlation loss for surgical video analysis” (127) (2020, 179 citations), and Yu's “Assessment of Automated Identification of Phases in Videos of Cataract Surgery Using Machine Learning and Deep Learning Techniques” (164) (2019, 106 citations) utilized surgical tool detection in workflow recognition. Nwoye's “Weakly supervised convolutional LSTM approach for tool tracking in laparoscopic videos” (101) (2019, 144 citations) utilized instrument segmentation to achieve accurate and reliable tool detection and tracking.

### 3.11 Top recent cited papers

The 3 most cited articles from 2020 onwards in surgical video analysis are shown in Table 6. Out of the 3 articles, 2 were published in ScienceDirect Elsevier and 1 in IEEE. The most highly cited article, Jin, “Multi-task recurrent convolutional network with correlation loss for surgical video analysis,” (127) was published by ScienceDirect (Elsevier), with 179 citations. The paper talks about surgical video analysis, focusing on tool detection and phase recognition. Jin and his co-authors were able to achieve such task through the utilization of CNN and RNNs to maintain the temporal elements. Next, Kitaguchi published the second most cited article during the last five years at 99, titled “Automated laparoscopic colorectal surgery workflow recognition using artificial intelligence” (165). The paper introduces a new dataset “LapSig300” which encompasses 300 intraoperative videos that were collected from 19 high-volume centers. This novel work describes the dataset components such as surgical workflows that were classified into nine phases, 3 actions and 5 tools. Other notable contributions during this recent timeframe include Jin's (2021) “Temporal Memory Relation Network for Workflow Recognition” (125) with 81 citations. The work presents a novel network that detects the surgical phase based on the presence of surgical tools by utilizing LSTM that preserves temporal information.

**Table 6:** The three most cited articles in the field from 2020 onwards, alongside their year of publication, primary author, total citations, and publisher.

Title	Year	Primary author	Total citations	Publisher	Application	DL Algorithms
Multi-task recurrent convolutional network with correlation loss for surgical video analysis (127)	(2020)	Jin	179	ScienceDirect ElSevier	Tool detection and phase recognition tasks	RNN and CNN
Automated laparoscopic colorectal surgery workflow recognition using artificial intelligence: Experimental research (165)	(2020)	Kitaguchi	99	ScienceDirect ElSevier	Tool detection, segmentation and surgical phase recognition	CNN
Temporal Memory Relation Network for Workflow Recognition From Surgical Video (125)	(2021)	Jin	81	IEEE	Tool presence and phase recognition	ResNet and LSTM

The number of citations were verified using Google Scholar.

Additionally, a growing subset of recent papers explores Transformer-based models and multimodal learning approaches, signaling a shift from purely convolutional or recurrent architectures toward methods capable of integrating video, textual, and even sensor data for more robust surgical context understanding.

## 4 Discussion

This bibliometric analysis demonstrates sustained growth and increasing global engagement in deep learning methods for surgical instrument perception in MIS. Instrument detection, segmentation, and tracking are key enabling technologies for broader surgical data science applications, including workflow recognition, skill assessment, and context-aware intraoperative assistance. The literature initially focused on CNN-based approaches and benchmark challenge datasets (e.g., EndoNet-era laparoscopic video recognition and the EndoVis series), but recent work increasingly explores transformer architectures and large-scale pretrained models. For example, Segment Anything–style models have begun to be adapted for robotic instrument segmentation [e.g., Surgical-DeSA M (10)], highlighting the emerging role of foundation-model paradigms in MIS. At the same time, progress remains dependent on high-quality annotated datasets and standardized benchmarking (e.g., EndoVis, HeiChole, Cholec80, CaDIS), alongside rigorous evaluation under domain shift, occlusion, smoke/bleeding artifacts, and strict real-time constraints (113). Overall, these trends suggest a maturing field moving toward more generalizable and clinically deployable solutions.

The sustained research interest seen in 2024 suggests that this field will likely continue to keep evolving, bringing further innovations to clinical practices. The field of DL in MIS is rapidly advancing, with a diverse range of countries, institutions, and authors contributing to its growth. Nevertheless, researchers are collaborating not only on a local level between institutions but also from different continents. The increasing number of publications and citations reflects the high relevance of this research in enhancing surgical outcomes. As more collaborative efforts arise and the technology advances, DL has the potential to transform surgical practices, enhancing both training and the precision and safety of procedures. The geographic distribution of publications shows that many countries are making significant contributions. There is a noticeable difference in how productive and impactful these contributions are

### 4.1 Leading countries in the field

**Figure 6:**
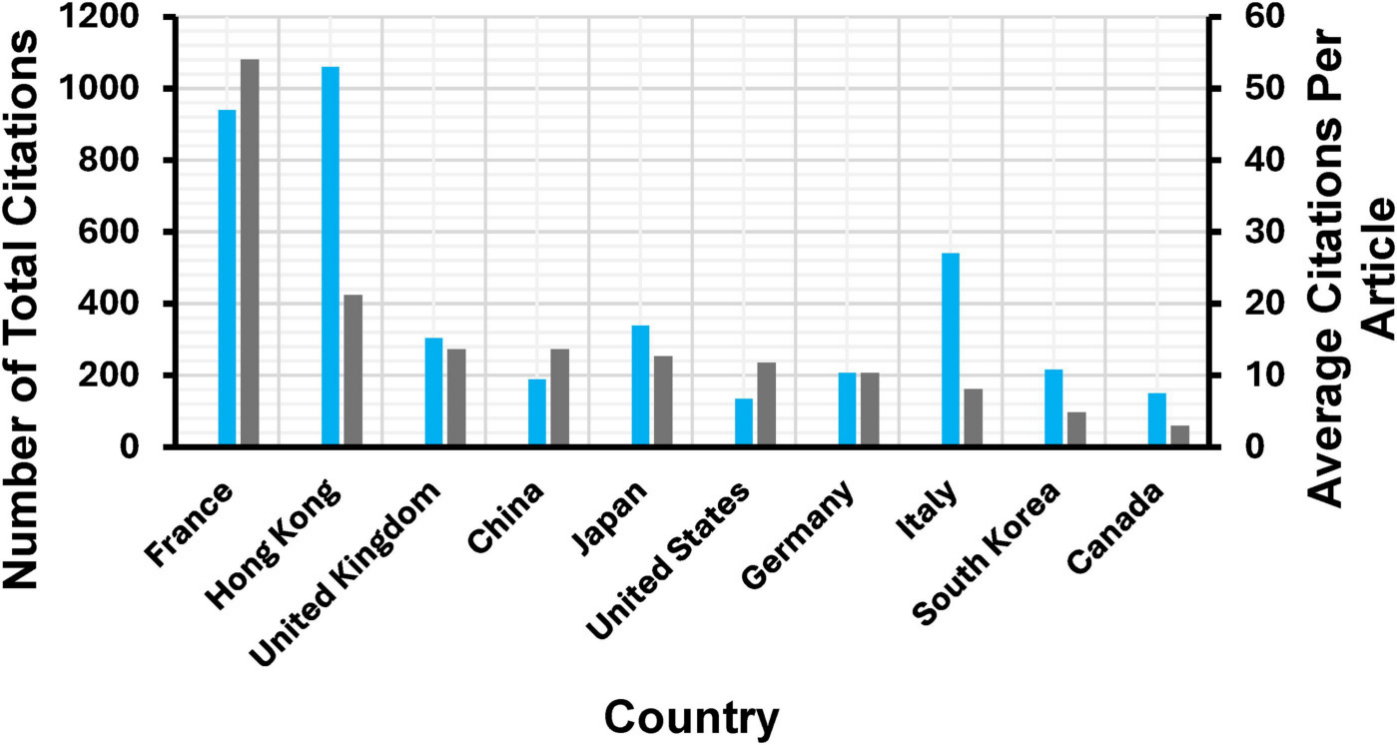
Comparison of the total number of citations and the average citations per article across various countries in DL research for surgical instrument segmentation in MIS. The gray bars represent the total number of citations (left axis), while the blue bars indicate the average citations per article (right axis).

**Figure 7:**
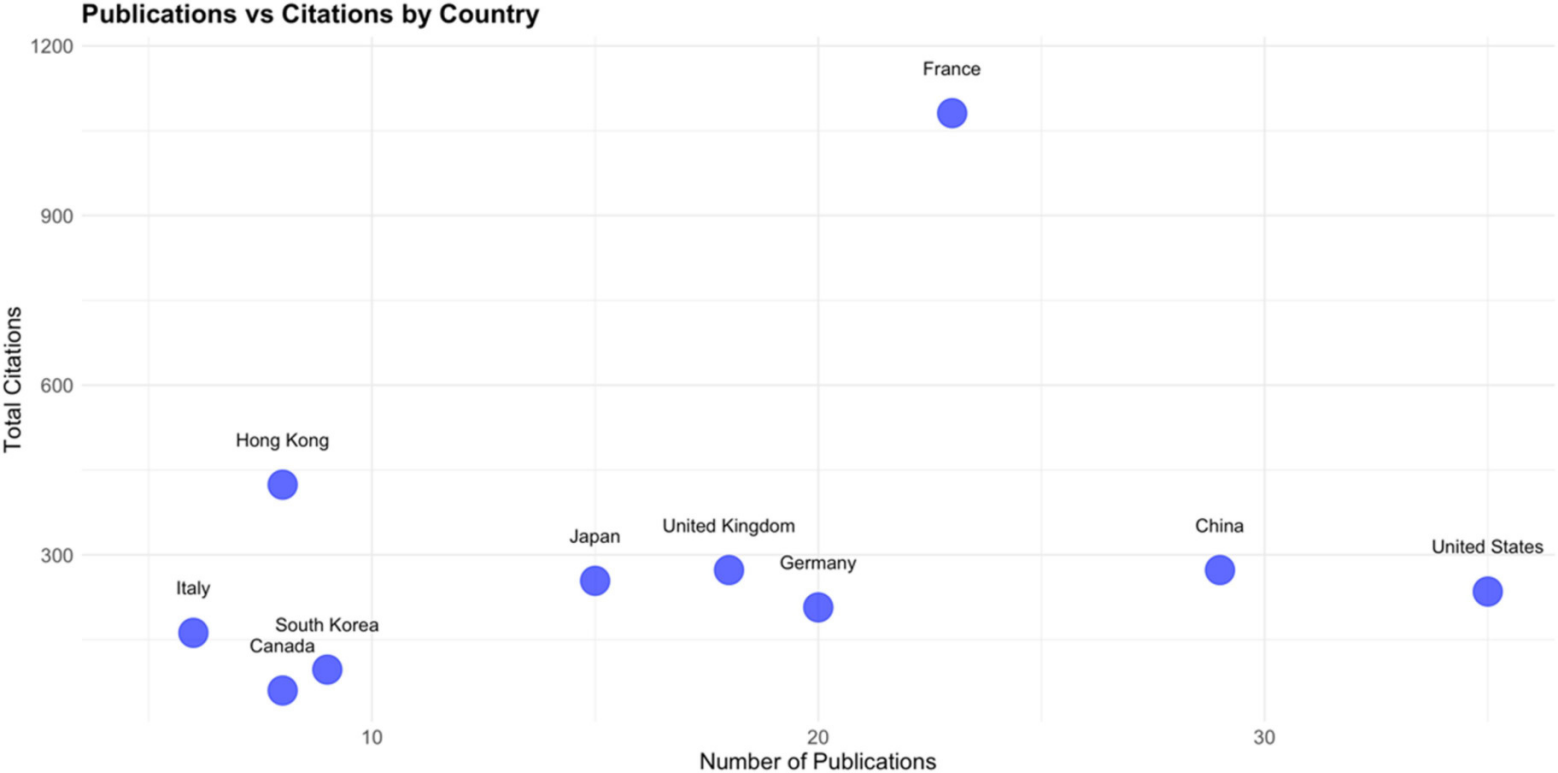
Total citation vs. number of publications for each country.

A comparative analysis of the total number of citations and the average citations per article offers valuable information into the balance between research volume and individual article influence in each country. Figure 6 highlights France as an outlier, with the highest citation count despite not leading in the number of publications. This indicates that France produces a high volume of influential research as the average citations per article is high (Figure 7). Hong Kong follows with fewer total citations compared to France, but still significant in number. Importantly, Hong Kong stands out with the highest average citations per article, indicating that while the number of publications may be lower, the impact of each publication is very high. This shows that the research from institutions in Hong Kong is highly targeted and impactful within its academic field, possibly reflecting a focus on quality over quantity. The UK shows a moderate number of total citations, only slightly behind Hong Kong. Its average citations per article are similarly high, reflecting that although the influence may be less, it is consistent throughout publications. China and Japan display similar trends with moderate total citations. Interestingly, their average citations per article are lower, implying that while these countries may publish frequently, the individual impact of each publication tends to be smaller. Despite the large quantities of research, the overall influence of each article may not be as pronounced.

Despite the reputation of the US in global research, the total number of citations is somewhat lower than expected, considering it has the highest number of publications per country. It has a modest average citation rate per article, showing that the country produces a steady stream of research, but its per-article influence is not as strong as other countries. This may warrant a deeper look into the quality or visibility of US publications in this research area. With both a low total citation count and a modest average citation rate, Germany's overall contribution appears less prominent. However, its consistent output may indicate steady participation in the field without significant high-impact publications. The citations on publications from Italy portray an interesting pattern. Though not among the highest in total citations, Italy's publications have a strong average citation per article, suggesting that its research, while less frequent, tends to be highly impactful. This suggests a focus on producing fewer but higher-quality or more widely recognized studies. Finally, both South Korea and Canada are on the lower end in terms of total citations and average citations per article. This shows that their contributions are either newer or less widely recognized in this field. This could also reflect limited participation or a developing research presence in the field.

However, on an interpretative note, we need to be cognizant of the fact that citation-based indicators reflect visibility and uptake of publications and are influenced by publication age, venue, and citation practices. Accordingly, differences in total citations and average citations per article should not be interpreted as direct measures of methodological quality or clinical effectiveness.

### 4.2 Country co-authorship

In terms of geographical distribution, the United States occupies a central position in the co-authorship network, characterized by a high number of international collaboration links. Many countries demonstrate frequent co-authorship with the United States, reflecting its central role in the network. France, despite a lower publication volume, shows a comparatively higher average citation rate per article, suggesting a strong impact of its research output. China has also shown rapid growth in both publications and citations, indicating its increasing influence in the field.

Further, institutions such as the University of Strasbourg, Furtwangen University, and Johns Hopkins University are at the forefront of DL research in MIS. This highlights how these institutions can drive the collection of information. Institutions from Europe, North America, and Asia account for the majority of publications, indicating broad geographic representation across these regions. However, collaboration involving institutions from underrepresented regions appears less frequent in the co-authorship network. Additionally, author and institutional collaboration networks demonstrate frequent connections within certain clusters, particularly between European, American, and East Asian countries. Authors like Stoyanov, Padoy, and Heng are central figures in this collaborative environment, driving forward impactful research. There is no doubt still room for growth in terms of incorporating more diverse contributors from underrepresented regions, which could lead to new perspectives and novel advancements.

**Figure 8:**
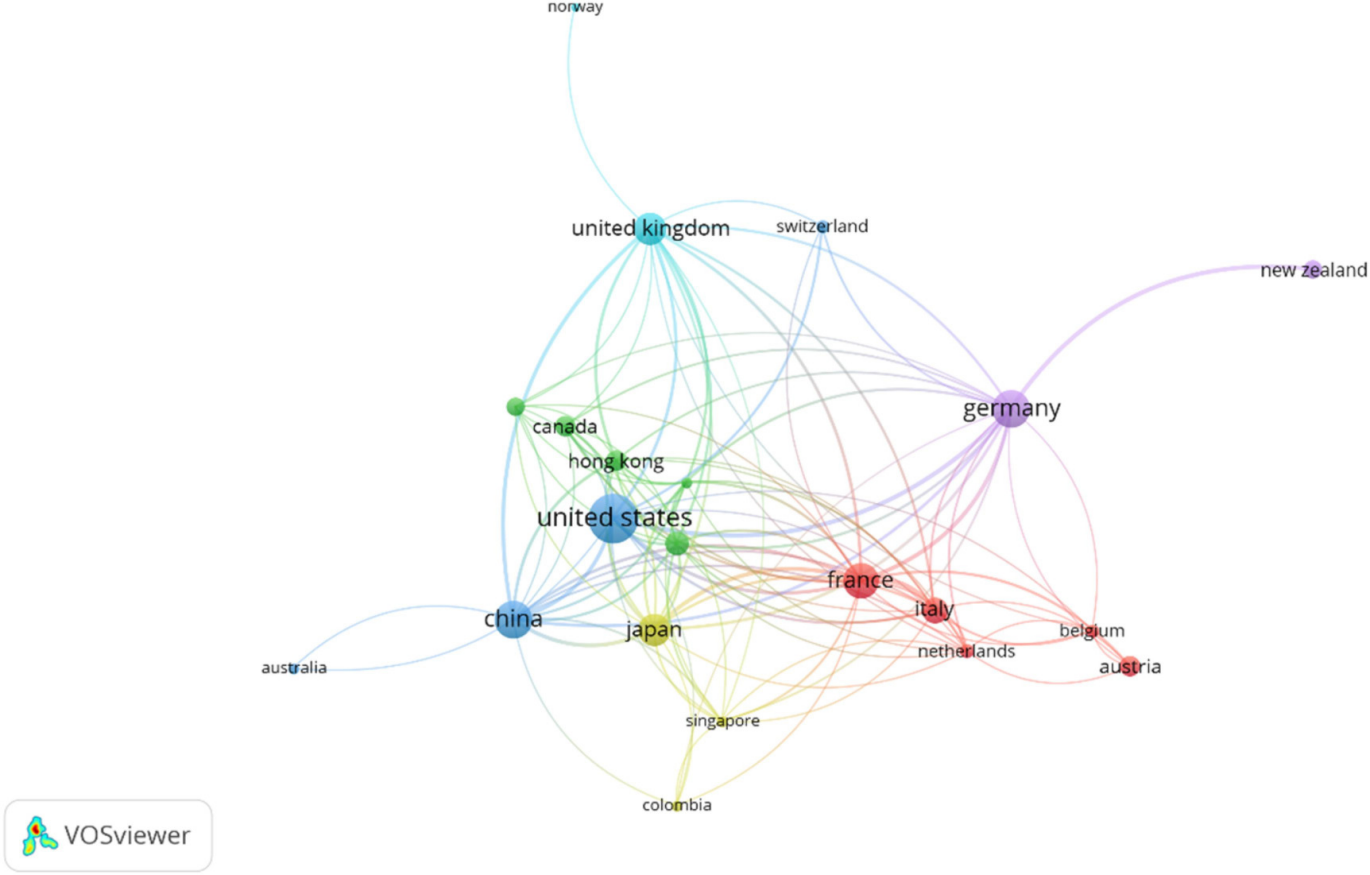
Co-authorship collaboration network of publications by country. The size of each country's circle represents the number of publications they have contributed, and the thickness of the lines represents the number of collaborations between the countries. The minimum number of article contributions for a country to be included in this network was set to 2. VOSviewer was used in the production of this figure.

The collaboration network in these publications, which focuses on several countries, displays a thorough, but unevenly distributed, structure of global cooperation (Figure 8). The United States demonstrates the highest number of co-authorship links with countries in Europe and East Asia. Several European countries, including the United Kingdom, Germany, and France, show frequent co-authorship links with institutions based in the United States. Similarly, East Asian countries like China, Japan, and South Korea also exhibit strong collaborative research both within the region and with the US. Furthermore, while other countries, like India, Australia, and Canada make significant contributions, their collaborative networks are not as extensive or dense as those seen in Europe and East Asia, indicating that there is room for growth and integration.

Despite active research clusters in Europe, North America, and East Asia, participation from regions such as Africa and Latin America remains limited, highlighting the need for more inclusive global collaborations and funding initiatives to broaden the field's impact. There are a variety of reasons why certain authors or countries may be isolated. It is possible that the author's work might not be widely disseminated or accessible to the broader research community. This can be the result of publication in less prominent journals, or even limited participation in international conferences and networks. Additionally, if the author's work spans across multiple disciplines, it might not fit neatly into the established research networks of any single field, leading to isolation within both publication and citation networks. Further, geographical or institutional factors might limit the author's opportunities for collaboration. Over time, as the field develops, there may be an increase in both collaborations and citations across authors from different countries and institutions over time.

### 4.3 Author collaboration and co-citation networks

**Figure 9:**
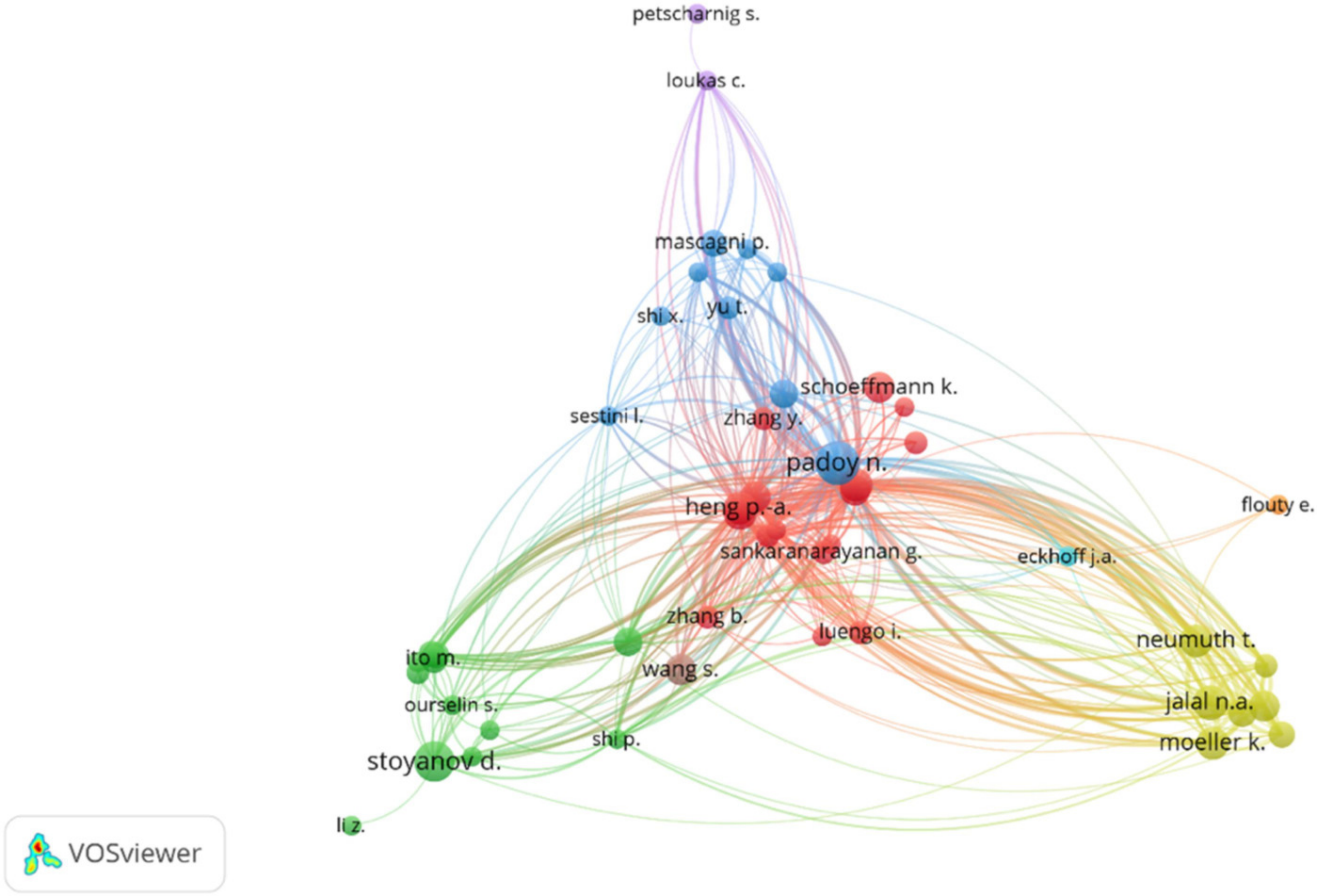
Author co-citation network of the included publications. The size of each author's circle represents the number of citations they have received, and the thickness of the lines represents the number of co-citations between the authors. The minimum number of citations for an author to be included in this network was set to 10. VOSviewer was used in the production of this figure.

The co-citation network represents the relationships between authors based on how often they are cited together in these scientific publications (Figure 9). Padoy appears to be a central figure with a lot of connections, which suggests that he is highly influential and widely cited across different studies. Additionally, Stoyanov and Jannin also appear to be very important in their clusters, demonstrating substantial impact and regular co-citation with other authors. Some authors like Jalal, who were found to be isolated in publications networks, were also found to be isolated in the citation networks. Inter-cluster connections suggest that influential authors are not only leaders within their specific areas but also contribute to interdisciplinary research, bridging gaps between different fields and fostering collaboration.

**Figure 10:**
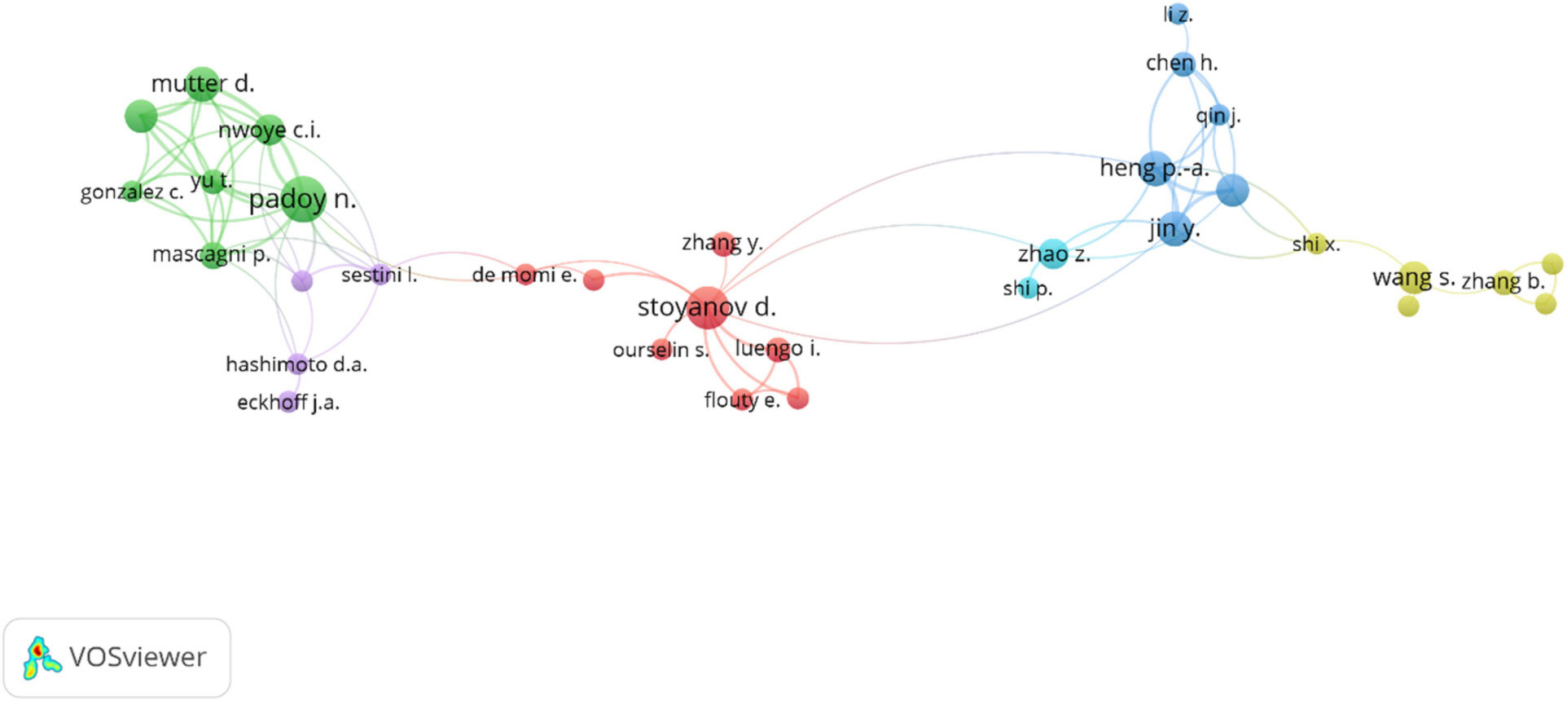
Co-authorship collaboration network of the publications in the field. The size of each author's circle represents the number of publications they have contributed, and the thickness of the lines represents the number of collaborations between the authors. The minimum number of article contributions for an author to be included in this network was set to 3. VOSviewer was used in the production of this figure.

Stoyanov plays a central role in collaborations, followed by Padoy and Heng, who act as bridges between various researchers (Figure 10). Author collaborations indicate high-impact research, with broadening opportunities for further collaboration. Given his extensive connections with other scholars, Stoyanov appears to have a crucial role in promoting cooperation both inside and outside of his cluster. Moreover, Padoy and Heng exhibit a noteworthy collaborative influence, serving as a bridge between various researchers. Certain researchers, like Loukas and Ito, seem to be more isolated with fewer connections.

### 4.4 Keyword co-occurrence analysis

The frequent occurrence of terms like “Deep Learning,” “Laparoscopy,” and “Convolutional Neural Networks” highlights the focus on laparoscopic surgeries as a key area for DL (Table 4). From these keywords, it is evident that the use of video data as opposed to images for training DL models is most common. Additionally, most DL models utilize CNNs in their architectures, given that it is the most frequent algorithm in the keyword analysis. This indicates that they have the DL algorithm with the most extensive application in image recognition and segmentation tasks. Additionally, Tables 5, 7 illustrate CNNs' popularity as they are widely utilized by the top cited papers in the field. The growing trend in the use of video-based data for DL training, as opposed to static images, is indicative of the shift towards its applications in surgery. This suggests that the future of DL in MIS may lie in improving intraoperative decision-making by providing real-time analysis and feedback to surgeons. This significant increase also aligns with the rapid technological advancements in DL algorithms and their increasing use in medical imaging and surgical robotics. It is clear that this is a developing field, as indicated by the rapid growth of publications and keyword use. By analyzing keywords in these research papers, important information can be acquired about research trends and focus areas in the field of DL use in MIS.

**Table 7:** Top ten journals with the highest number of articles published in the field, alongside their impact factors from the 2022 journal impact factors by annualreviews.org.

Source	Number of Articles	IF 2022
International Journal of Computer Assisted Radiology and Surgery	27	2.3
Lecture Notes in Computer Science	19	1.1
Medical Image Analysis	11	10.9
IEEE Transactions on Medical Imaging	10	10.6
Surgical Endoscopy	10	2.4
Current Directions in Biomedical Engineering	9	0.5
IEEE Robotics and Automation Letters	4	4.6
International Journal of Medical Robotics and Computer Assisted Surgery	4	2.3
Computer Methods in Biomechanics and Biomedical Engineering: Imaging and Visualization	4	1.3
Computer Methods and Programs in Biomedicine	3	4.9

Conference proceedings were excluded from this table.

**Figure 11:**
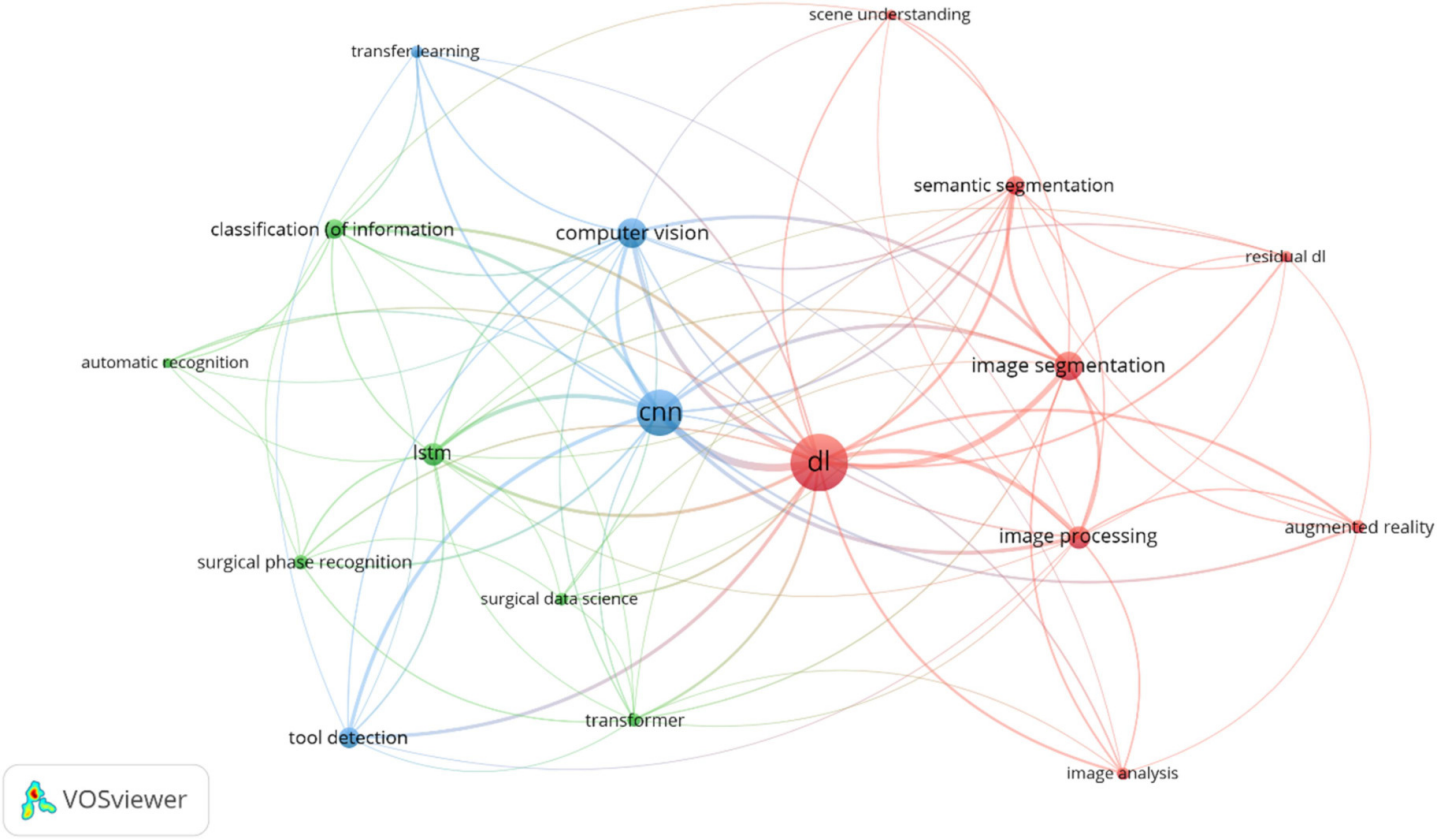
Data science and deep learning keyword co-occurrence network of the included publications. The size of each keyword's circle represents the number of publications they have occurred in, and the thickness of the lines represents the number of co-occurrences between the keywords. The minimum number of occurrences for a keyword to be included in this network was set to 5. VOSviewer was used in the production of this figure.

The keyword co-occurrence network in Figure 11 provides insights into key technical terms and their relationships in DL. The central term, “DL,” is closely linked with “image segmentation,” “computer vision,” “CNN,” and “AI,” reflecting the focus on DL techniques for image analysis in surgery. The green cluster highlights terms such as “image segmentation,” “image processing,” and “semantics,” which highlights some of the most commonly used techniques by DL algorithms in the processing of surgical videos. These models are critical for accurately tracking surgical instruments and automating workflow recognition. The blue cluster features “CNN” and “LSTM” (long short-term memory networks), suggesting that both spatial and temporal features are handled through a combination of neural networks. These tools enhance real-time surgical decision-making by identifying various phases of surgery. Lastly, the purple cluster emphasizes “object recognition” and “instrument detection,” addressing the importance of these tasks in developing a function DL algorithm. Recent works are using transformers, however, as it is till new to the field of surgical video analysis it less frequent given that the timeframe is since 2017, where CNNs were dominating the field.

**Figure 12:**
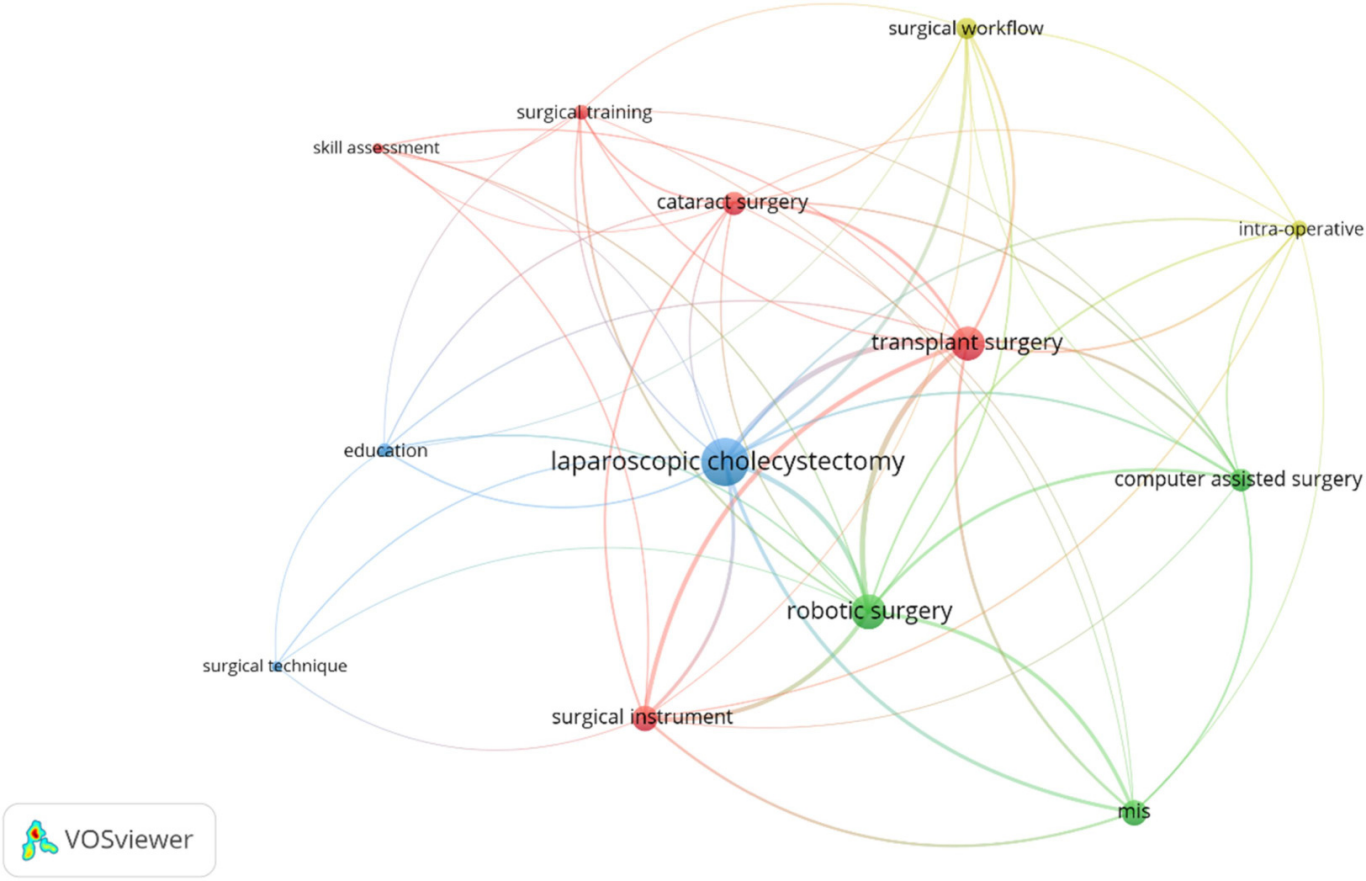
Surgery related keyword co-occurrence network of the included publications. The size of each keyword's circle represents the number of publications they have occurred in, and the thickness of the lines represents the number of co-occurrences between the keywords. The minimum number of occurrences for a keyword to be included in this network was set to 5. VOSviewer was used in the production of this figure.

The keyword co-occurrence network in Figure 12 is more focused on surgical and medical terminology, illustrating their relationships to MIS. The central node, “surgical equipment,” is closely connected to multiple terms such as “robotic surgery,” “endoscopic surgery,” and “laparoscopic cholecystectomy,” indicating the DL algorithms used had a particular focus on instrumentation in all these types of surgeries. The green cluster, which includes terms like “surgical instrument” and “surgical tools,” highlights the focus on instrument development and refinement, which is essential for enhancing precision and outcomes in MIS procedures. The connection to “transplant surgery” shows that advancements in surgical tools are not just limited to one type of surgery but also seem to have broad applications across different fields. In the red cluster, terms such as “medical imaging” and “neurosurgery” emphasize the integration of imaging techniques in surgeries, especially for complex procedures like brain surgery. This cluster demonstrates the critical role of imaging in guiding surgical procedures and improving surgical accuracy. The link to “surgical phase recognition” suggests the growing importance of recognizing and automating phases of surgery using advanced tools and DL models.

### 4.5 Popular deep learning models

DL is significantly contributing to the surgical domain, particularly through convolutional neural networks (CNNs). CNNs are highly effective for processing image data, making them popular for the integration with other algorithms, such as recurrent neural networks (RNNs) and long short-term memory networks (LSTMs), which preserve temporal features. This combination is beneficial when maintaining the temporal information across different frames is critical, like surgical workflow recognition.

Recurrent networks are extensively utilized in tracking applications, such as tool tracking, where they maintain continuity by connecting subsequent frames and preventing the loss of recognized or split items. However, tasks that are within the spatial components only can be achieved using CNNs only like classification, localization, or segmentation of surgical instruments. Notably, Encoder-decoder architecture is suitable for generating segmentation masks, including binary, semantic, or tool parts. In this network, the encoder identifies and captures important features, then the decoder reconstructs them into an abstract form.

Three-dimensional CNNs are used to identify surgical tool joints as they harness data from the third dimension. Moreover, numerous articles utilize existing CNN-based models such as YOLOv3 and ResNet, illustrating the adaptability of CNNs across diverse architectural frameworks to tackle unique issues in surgical operations.

### 4.6 Detection, segmentation and tracking applications

Within our corpus, instrument perception research in MIS can be grouped into three closely related tasks: detection (tool presence and coarse localization), segmentation (pixel-level delineation), and tracking/pose estimation (temporal continuity and articulation). Earlier high-impact studies frequently emphasized detection and tool presence classification, often as components of workflow analysis and training pipelines (166) where coarse spatial cues are sufficient, and were commonly evaluated on public laparoscopic video datasets such as Cholec80 (50).

More recent publications increasingly focus on segmentation, driven by the need for precise spatial understanding and by the maturation of benchmark datasets and challenges. Studies published include approaches that leverage transformer backbones and foundation-model paradigms for robust instrument segmentation under occlusion and scene variability, such as for example, adaptations of Segment Anything–style models for robotic instrument segmentation on EndoVis benchmarks [e.g., Surgical-DeSAM (10)]. Segmentation outputs are also being integrated into downstream applications such as objective performance assessment and context-aware assistance (167).

Tracking and pose estimation extend detection and segmentation across video frames and support higher-level scene understanding by enforcing temporal consistency. Recent spatio-temporal architectures have been used to track instrument motion (31) and articulation (163) and to support phase/step recognition, with benchmarking on datasets such as Cholec80 and EndoVis. Together, these complementary task families suggest a continued shift toward unified, real-time models that jointly model spatial detail and temporal dynamics.

### 4.7 Content analysis of research topics and subfields within surgical video analysis

The top ten most cited papers show the use of DL algorithms for a wide variety of purposes, including in MIS (Table 5). Twinada's “EndoNet: A Deep Architecture for Recognition Tasks on Laparoscopic Videos” (6) introduced a very popular convolutional neural network architecture named EndoNet. Although it focuses on surgical phase recognition in a variety of surgeries, it has only been applied to laparoscopic surgeries in this study. Instrument segmentation is crucial for this process, enabling the accurate tracking of instruments and allowing the algorithm to recognize the current phase of the surgery. Similarly, Jin's 2018 article “SV-RCNet: Workflow Recognition From Surgical Videos Using Recurrent Convolutional Network” (7) also focuses on workflow recognition, specifically in laparoscopic cholecystectomy videos. Jin's approach combines a convolutional neural network and a recurrent neural network, allowing for better utilization of both the temporal and visual features present in surgical videos. Padoy's 2019 (162) and Dergachyova's 2016 articles also focus on similar objective, using neural networks and other DL algorithms to identify the various stages of surgical procedures and the utilization of endoscopic videos for the training of these algorithms.

Six out of the 10 most cited articles were published in IEEE, likely because the field was in its early stages of development and the research was more technical in nature. At that time, the focus was on algorithms and specific technology, which was not clinically applicable yet. As the field progressed and deep learning applications became more integrated into surgical practice, the research gained clinical relevance. This has led to an increase in publications in medical journals rather than the more technical journals like the ones published by IEEE. This shift reflects the growing importance of these technologies to surgeons as they transitioned from experimental to practical, clinical tools.

## 5 Limitations of this study

Despite the comprehensive nature of this bibliometric analysis, several limitations should be acknowledged. First, restricting the search strategy to six major databases may have led to the exclusion of relevant studies indexed elsewhere or published in non-English languages. As with any bibliometric analysis, the observed patterns are shaped by the structure and indexing practices of the selected databases and therefore reflect trends within the indexed academic literature rather than the entirety of research activity in this domain. Additionally, the reliance on specific search terms (e.g., “deep learning,” “robotic-assisted surgery,” “minimally invasive surgery”) could have omitted certain papers that used alternative terminologies or did not explicitly mention deep learning in the title or abstract. Second, citation-based metrics can be influenced by factors such as varying publication times and self-citations, potentially skewing the apparent impact of certain studies. Third, conference proceedings that are often pivotal for rapidly evolving fields like deep learning may be underrepresented if they were not consistently indexed. Moreover, limiting the scope to original research involving deep-learning models for instrument segmentation, detection, or tracking may overlook valuable insights from review articles, meta-analyses, or purely simulation-based studies. Moreover, bibliometric analyses characterize quantitative and relational patterns in the literature and do not assess the methodological quality, technical rigor, or clinical relevance of individual studies. Importantly, while co-authorship and co-citation networks illuminate collaboration patterns, they do not capture the quality of these partnerships or the nuances of data sharing and methodological reproducibility across different research groups. Finally, because this is a bibliometric analysis, it characterizes research activity and influence rather than technical performance or patient outcomes; therefore, implications for clinical practice should be interpreted indirectly.

## 6 Future direction and challenges

Building on the current trends observed in DL for MIS, several avenues appear especially promising. As the field transitions from experimental algorithms to clinical deployment, explainable AI and regulatory frameworks will become essential for ensuring transparency and patient safety. Real-time analysis potentially facilitated by edge computing and high-efficiency models can significantly advance intraoperative guidance, allowing surgeons to receive immediate, context-aware feedback.

An emerging direction is the development of agentic AI solutions for the operating room in the form of software agents that can maintain a structured representation of the evolving surgical context (e.g., surgical phase, instruments, anatomy, and patient status) and plan multi-step assistance actions. Rather than producing single-task predictions in isolation, an agentic system could integrate outputs from perception modules (instrument/scene segmentation and tracking), workflow recognition, device telemetry, and peri-operative data streams. This could potentially enable proactive, context-aware decision support such as tailored safety-check prompts, anomaly-detection alerts, and guideline-consistent “next-step” suggestions, while also streamlining routine tasks such as intraoperative documentation and equipment-readiness checks. However, because such agents may initiate recommendations without explicit prompting, safe deployment will require careful human-in-the-loop design, transparent uncertainty reporting, audit trails, rigorous prospective validation, and clear governance around cybersecurity, privacy, and accountability to ensure these systems support (rather than replace) clinician judgement.

Moreover, multimodal learning (combining visual data with text, sensor data, or electronic health records) stands to enrich surgical decision-making, while foundation models pretrained on large-scale datasets could be fine-tuned for MIS-specific tasks. Recent work illustrates how language–vision pretraining can be adapted for surgical scene understanding [e.g., CLIP-based approaches for surgical scene segmentation (168)]. In parallel, there is growing momentum toward holistic surgical scene segmentation that jointly delineate instruments and relevant anatomy which may better support context-aware assistance and downstream analytics than tool-only models (169, 170). Concurrently, there is also a growing need for standardized datasets and labeling protocols, which will enhance reproducibility and reduce the fragmentation currently seen in surgical video annotation efforts. Beyond intraoperative deployment, these perception capabilities can also be leveraged in simulation-based education and skills assessment—where interface design and serious-game/gamified training environments may benefit from reliable instrument/scene understanding (171, 172). On the international front, broadening collaboration across underrepresented regions could diversify research perspectives and spur innovations that are globally relevant. Finally, continued integration with robotic platforms could pave the way for semi-autonomous or even fully autonomous surgical maneuvers, provided safety and ethical concerns are rigorously addressed.

## 7 Conclusions

This bibliometric analysis demonstrates the rapid growth and increasing maturity of deep learning research in minimally invasive surgery. Our analysis revealed that research in this was field was globally distributed but concentrated in certain regions, institutions, and authors. Early advances in instrument detection and surgical workflow analysis were followed by sustained increases in publication volume and citation impact, particularly across Europe, North America, and East Asia. Several regions, including France, Hong Kong, and the United Kingdom, exhibit high citation influence relative to output, indicating that impact is driven by research quality and network position rather than volume alone. The rising prominence of deep learning and laparoscopy-related keywords reflects continued emphasis on video-based intraoperative analysis. Structural limitations were also noticed, including fragmented datasets and inconsistent benchmarking, which constrain reproducibility and cross-study comparability. Future progress in DL for MIS will depend on clinically deployable, explainable, and real-time methods supported by standardized data and broader collaboration. Continued integration with multimodal inputs and surgical platforms will be critical to ensure safe and effective translation into practice.

Overall, this review delineates how deep learning research in minimally invasive surgery has evolved and where influence and structural limitations persist. Emerging architectures like Transformers, the growing emphasis on explainability, and the adoption of large, community-driven datasets suggest that the field is poised for another phase of innovation. By mapping the existing research landscape and identifying influential studies, contributors, and collaborations, it provides an empirical basis to guide future research directions, optimize inter-institutional partnerships, and drive meaningful contributions to the field of DL in MIS.

## Data Availability

The original contributions presented in the study are included in the article/Supplementary Material, further inquiries can be directed to the corresponding author.

## References

[1] Blencowe NS Waldon R Vipond MN. Management of patients after laparoscopic procedures. Br Med J. (2018) 360:k120. 10.1136/bmj.k120 29437677

[2] Sheetz KH Claflin J Dimick JB. Trends in the adoption of robotic surgery for common surgical procedures. JAMA Netw Open. (2020) 3:e1918911. 10.1001/jamanetworkopen.2019.18911 31922557 PMC6991252

[3] Von Ahlen C Geissler A Vogel J. Comparison of the effectiveness of open, laparoscopic, and robotic-assisted radical prostatectomies based on complication rates: a retrospective observational study with administrative data from Switzerland. BMC Urol*.* (2024) 24:215. 10.1186/s12894-024-01597-3 39375695 PMC11457412

[4] Allan M Shvets A Kurmann T Zhang Z Duggal R Su Y-H 2017 Robotic Instrument Segmentation Challenge. (2019). 10.48550/ARXIV.1902.06426

[5] Bodenstedt S Rivoir D Jenke A Wagner M Breucha M Müller-Stich B Active learning using deep Bayesian networks for surgical workflow analysis. Int J CARS. (2019) 14:1079–87. 10.1007/s11548-019-01963-9 30968355

[6] Twinanda AP Shehata S Mutter D Marescaux J de Mathelin M Padoy N. Endonet: a deep architecture for recognition tasks on laparoscopic videos. IEEE Trans Med Imaging. (2017) 36:86–97. 10.1109/TMI.2016.2593957 27455522

[7] Jin Y Dou Q Chen H Yu L Qin J Fu C-W SV-RCNet: workflow recognition from surgical videos using recurrent convolutional network. IEEE Trans Med Imaging. (2018b) 37:1114–26. 10.1109/TMI.2017.2787657 29727275

[8] Padoy N. Machine and deep learning for workflow recognition during surgery. Minim Invasive Ther Allied Technol MITAT Off J Soc Minim Invasive Ther*.* (2019) 28:82–90. 10.1080/13645706.2019.1584116 30849261

[9] Kiyasseh D Ma R Haque TF Miles BJ Wagner C Donoho DA A vision transformer for decoding surgeon activity from surgical videos. Nat. Biomed Eng. (2023) 7:780–96. 10.1038/s41551-023-01010-8 PMC10307635 36997732

[10] Sheng Y Bano S Clarkson MJ Islam M. Surgical-DeSAM: decoupling SAM for instrument segmentation in robotic surgery. Int J Comput Assist Radiol Surg. (2024) 19:1267–71. 10.1007/s11548-024-03163-6 38758289 PMC11230981

[11] Marzullo A Moccia S Catellani M Calimeri F Momi ED. Towards realistic laparoscopic image generation using image-domain translation. Comput Methods Programs Biomed*.* (2021) 200:105834. 10.1016/j.cmpb.2020.105834 33229016

[12] Wang R Zhang D Li Q Zhou X-Y Lo B. Real-time surgical environment enhancement for robot-assisted minimally invasive surgery based on super-resolution. arXiv [Preprint]. arXiv:2011.04003 (2020). 10.48550/ARXIV.2011.04003

[13] D’Andria Ursoleo J Losiggio R Monaco F. The first 100 years of the British journal of anaesthesia: a bibliometric analysis of the top-cited articles. Br J Anaesth. (2024) 132:789–92. 10.1016/j.bja.2024.01.016 38336514

[14] Akmal M Hasnain N Rehan A Iqbal U Hashmi S Fatima K Glioblastome Multiforme: a bibliometric analysis. World Neurosurg. (2020) 136:270–82. 10.1016/j.wneu.2020.01.027 31953095

[15] Bidwe RV Mishra S Patil S Shaw K Vora DR Kotecha K Deep learning approaches for video compression: a bibliometric analysis. Big Data Cogn Comput. (2022) 6:44. 10.3390/bdcc6020044

[16] Ursoleo JD Bottussi A Christiansen S Sullivan DR Vranas KC Rosa WE Mapping the science of palliative care: a bibliometric analysis of the top 100 cited articles. Palliat Med. (2025a) 39:922–35. 10.1177/02692163251362560 40842428 PMC12476462

[17] D’Andria Ursoleo J Calì C Losiggio R Limone V Mucci E Monaco F. Spiritual care in palliative medicine and End of life: a bibliometric network analysis. J Palliat Med. (2025b) 28:265–79. 10.1089/jpm.2024.0007 39093919

[18] D’Andria Ursoleo J Losiggio R Licheri M Barucco G Lazzari S Faustini C Minimal invasive extracorporeal circulation: a bibliometric network analysis of the global scientific output. Perfusion. (2025c) 40:1176–86. 10.1177/02676591241269729 39288245 PMC12202833

[19] Li D Yu D Li Y Yang R. A bibliometric analysis of PROTAC from 2001 to 2021. Eur J Med Chem*.* (2022) 244:114838. 10.1016/j.ejmech.2022.114838 36274273

[20] Li Z Maimaiti Z Fu J Chen J-Y Xu C. Global research landscape on artificial intelligence in arthroplasty: a bibliometric analysis. Digit Health. (2023) 9:20552076231184048. 10.1177/20552076231184048 37361434 PMC10286212

[21] Wang H Shi J Shi S Bo R Zhang X Hu Y. Bibliometric analysis on the progress of chronic heart failure. Curr Probl Cardiol. (2022) 47:101213. 10.1016/j.cpcardiol.2022.101213 35525461

[22] Aria M Cuccurullo C. Bibliometrix: an R-tool for comprehensive science mapping analysis. J Informetr*.* (2017) 11:959–75. 10.1016/j.joi.2017.08.007

[23] Van Eck NJ Waltman L. Software survey: VOSviewer, a computer program for bibliometric mapping. Scientometrics. (2010) 84:523–38. 10.1007/s11192-009-0146-3 20585380 PMC2883932

[24] Sahu M Mukhopadhyay A Szengel A Zachow S. Addressing multi-label imbalance problem of surgical tool detection using CNN. Int J Comput Assist Radiol Surg*.* (2017) 12:1013–20. 10.1007/s11548-017-1565-x 28357628

[25] Hu X Yu L Chen H Qin J Heng P-A AGNet: attention-guided network for surgical tool presence detection. In: Cardoso MJ Arbel T Carneiro G Syeda-Mahmood T Tavares JMRS Moradi M, editors. Deep Learning in Medical Image Analysis and Multimodal Learning for Clinical Decision Support. Cham: Springer International Publishing (2017). p. 186–94. 10.1007/978-3-319-67558-9_22

[26] Wang S Raju A Huang J. Deep learning based multi-label classification for surgical tool presence detection in laparoscopic videos. In: 2017 IEEE 14th international symposium on biomedical imaging (ISBI 2017) 620–3; IEEE, Melbourne, Australia; (2017). 10.1109/ISBI.2017.7950597

[27] Mishra K Sathish R Sheet D. Learning latent temporal connectionism of deep residual visual abstractions for identifying surgical tools in laparoscopy procedures. In: 2017 IEEE Conference on Computer Vision and Pattern Recognition Workshops (CVPRW) 2233–40; IEEE; Honolulu, HI, USA; (2017). 10.1109/CVPRW.2017.277

[28] Leibetseder A Primus MJ Petscharnig S Schoeffmann K. Real-time image-based smoke detection in endoscopic videos. In: Proceedings of the on thematic workshops of ACM multimedia 2017 296–304; ACM, Mountain View; California USA; (2017). 10.1145/3126686.3126690

[29] Al Hajj H Lamard M Conze P-H Roychowdhury S Hu X Maršalkaitė G CATARACTS: challenge on automatic tool annotation for cataRACT surgery. Med Image Anal. (2019) 52:24–41. 10.1016/j.media.2018.11.008 30468970

[30] Attia M Hossny M Nahavandi S Asadi H. Surgical tool segmentation using a hybrid deep CNN-RNN auto encoder-decoder. In: 2017 IEEE International Conference on Systems, Man, and Cybernetics (SMC) 3373–8; IEEE; Banff, AB; (2017). 10.1109/SMC.2017.8123151

[31] Hegde SR Namazi B Iyengar N Cao S Desir A Marques C Automated segmentation of phases, steps, and tasks in laparoscopic cholecystectomy using deep learning. Surg Endosc. (2024) 38:158–70. 10.1007/s00464-023-10482-3 37945709

[32] Ban Y Eckhoff JA Ward TM Hashimoto DA Meireles OR Rus D Concept graph neural networks for surgical video understanding. IEEE Trans Med Imaging. (2024) 43:264–74. 10.1109/TMI.2023.3299518 37498757

[33] Wang Y Wu Z Dai J Morgan TN Garbens A Kominsky H Evaluating robotic-assisted partial nephrectomy surgeons with fully convolutional segmentation and multi-task attention networks. J Robotic Surg. (2023) 17:2323–30. 10.1007/s11701-023-01657-0 PMC10492672 37368225

[34] Lou A Tawfik K Yao X Liu Z Noble J. Min-max similarity: a contrastive semi-supervised deep learning network for surgical tools segmentation. IEEE Trans Med Imaging. (2023) 42:2832–41. 10.1109/TMI.2023.3266137 37037256 PMC10597739

[35] King D Adidharma L Peng H Moe K Li Y Yang Z Automatic summarization of endoscopic skull base surgical videos through object detection and hidden markov modeling. Comput Med Imaging Graph. (2023) 108:102248. 10.1016/j.compmedimag.2023.102248 37315397

[36] Fischer E Jawed KJ Cleary K Balu A Donoho A Thompson Gestrich W A methodology for the annotation of surgical videos for supervised machine learning applications. Int J CARS. (2023) 18:1673–8. 10.1007/s11548-023-02923-0 37245179

[37] Hasan SMK Simon RA Linte CA. Inpainting surgical occlusion from laparoscopic video sequences for robot-assisted interventions. J Med Imaging. (2023) 10:045002. 10.1117/1.JMI.10.4.045002 PMC10462486 37649957

[38] Ding H Wu JY Li Z Unberath M. Rethinking causality-driven robot tool segmentation with temporal constraints. Int J Comput Assist Radiol Surg. (2023) 18:1009–16. 10.1007/s11548-023-02872-8 37027082

[39] Nespolo RG Cole E Wang D Yi D Leiderman YI. A platform for tracking surgeon and observer gaze as a surrogate for attention in ophthalmic surgery. Ophthalmol Sci. (2023) 3:100246. 10.1016/j.xops.2022.100246 36748062 PMC9898791

[40] Eckhoff JA Ban Y Rosman G Müller DT Hashimoto DA Witkowski E TEsonet: knowledge transfer in surgical phase recognition from laparoscopic sleeve gastrectomy to the laparoscopic part of ivor–lewis esophagectomy. Surg Endosc. (2023) 37:4040–53. 10.1007/s00464-023-09971-2 36932188 PMC10156818

[41] Zhang B Goel B Sarhan MH Goel VK Abukhalil R Kalesan B Surgical workflow recognition with temporal convolution and transformer for action segmentation. Int J CARS. (2022a) 18:785–94. 10.1007/s11548-022-02811-z 36542253

[42] Yang JH Goodman ED Dawes AJ Gahagan JV Esquivel MM Liebert CA Using AI and computer vision to analyze technical proficiency in robotic surgery. Surg Endosc. (2023) 37:3010–7. 10.1007/s00464-022-09781-y 36536082

[43] Yeh H-H Jain AM Fox O Sebov K Wang SY. Phacotrainer: deep learning for cataract surgical videos to track surgical tools. Transl Vis Sci Technol. (2023) 12:23. 10.1167/tvst.12.3.23 36947046 PMC10050900

[44] Zhang B Sturgeon D Shankar AR Goel VK Barker J Ghanem A Surgical instrument recognition for instrument usage documentation and surgical video library indexing. Comput Methods Biomech Biomed Eng Imaging Vis. (2023b) 11:1064–72. 10.1080/21681163.2022.2152371

[45] Markarian N Kugener G Pangal DJ Unadkat V Sinha A Zhu Y Validation of machine learning–based automated surgical instrument annotation using publicly available intraoperative video. Oper Neurosurg. (2022) 23:235–40. 10.1227/ons.0000000000000274 35972087

[46] Mao G Lubelski D Zakaria HM Theodore N. Image-guided minimally invasive surgery for treatment of the Bertolotti syndrome—a case study: 2-dimensional operative video. Oper Neurosurg*.* (2022) 22:e222–3. 10.1227/ons.0000000000000132 35426882

[47] Matton N Qalieh A Zhang Y Annadanam A Thibodeau A Li T Analysis of cataract surgery instrument identification performance of convolutional and recurrent neural network ensembles leveraging BigCat. Trans Vis Sci Tech. (2022) 11:1. 10.1167/tvst.11.4.1 PMC8976933 35363261

[48] Leifman G Aides A Golany T Freedman D Rivlin E. Pixel-accurate segmentation of surgical tools based on bounding box annotations. In: 2022 26th International Conference on Pattern Recognition (ICPR) 5096–103; IEEE; Montreal, QC, Canada (Tor); (2022). 10.1109/ICPR56361.2022.9956530

[49] Yang Z Simon R Linte CA. A weakly supervised learning approach for surgical instrument segmentation from laparoscopic video sequences. In: Linte CA Siewerdsen JH, editors. Medical Imaging 2022: Image-Guided Procedures, Robotic Interventions, and Modeling, Volume 60. San Diego, United States: SPIE (2022). p. 10.1117/12.2610778. 10.1117/12.2610778

[50] Namazi B Sankaranarayanan G Devarajan V. A contextual detector of surgical tools in laparoscopic videos using deep learning. Surg Endosc. (2022) 36:679–88. 10.1007/s00464-021-08336-x 33559057 PMC8349373

[51] Yeh H-H Jain AM Fox O Wang SY. Phacotrainer: a multicenter study of deep learning for activity recognition in cataract surgical videos. Transl Vis Sci Technol. (2021) 10:23. 10.1167/tvst.10.13.23 34784415 PMC8606857

[52] Lin S Qin F Peng H Bly RA Moe KS Hannaford B. Multi-Frame feature aggregation for real-time instrument segmentation in endoscopic video. IEEE Robot Autom Lett. (2021) 6:6773–80. 10.1109/LRA.2021.3096156

[53] Meireles OR Rosman G Altieri MS Carin L Hager G Madani A SAGES Consensus recommendations on an annotation framework for surgical video. Surg Endosc. (2021) 35:4918–29. 10.1007/s00464-021-08578-9 34231065

[54] Kelly JD Petersen A Lendvay TS Kowalewski TM. Bidirectional long short-term memory for surgical skill classification of temporally segmented tasks. Int J Comput Assist Radiol Surg. (2020) 15:2079–88. 10.1007/s11548-020-02269-x 33000365 PMC7677176

[55] Jain S Lee S Barber SR Chang EH Son Y-J. Virtual reality based hybrid simulation for functional endoscopic sinus surgery. IISE Trans Healthc Syst Eng*.* (2020) 10:127–41. 10.1080/24725579.2019.1692263

[56] Kim TS O'Brien M Zafar S Hager GD Sikder S Vedula SS. Objective assessment of intraoperative technical skill in capsulorhexis using videos of cataract surgery. Int J CARS. (2019) 14:1097–105. 10.1007/s11548-019-01956-8 30977091

[57] McTaggart MI Higgins WE. Robust video-frame classification for bronchoscopy. In: Fei B Linte CA, editors. Medical Imaging 2019: Image-Guided Procedures, Robotic Interventions, and Modeling, Volume 61. San Diego, United States: SPIE, (2019). p. 10.1117/12.2507290. 10.1117/12.2507290

[58] Wang S Xu Z Yan C Huang J. Graph convolutional nets for tool presence detection in surgical videos. In: Chung ACS Gee JC Yushkevich PA Bao S, editors. Information Processing in Medical Imaging, Volume 11492. Cham: Springer International Publishing (2019). p. 467–78.

[59] El-Haddad MT Malone JD Hoang NT Tao YK. Deep-learning based automated instrument tracking and adaptive-sampling of intraoperative OCT for video-rate volumetric imaging of ophthalmic surgical maneuvers. In: Izatt JA Fujimoto JG. Optical Coherence Tomography and Coherence Domain Optical Methods in Biomedicine XXIII, 57. San Francisco, United States: SPIE (2019). p. 10.1117/12.2511827. 10.1117/12.2511827

[60] Shvets AA Rakhlin A Kalinin AA Iglovikov VI. Automatic instrument segmentation in robot-assisted surgery using deep learning. In: 2018 17th IEEE international conference on machine learning and applications (ICMLA); 624–628; (2018). 10.1109/ICMLA.2018.00100

[61] Jin A Yeung S Jopling J Krause J Azagury D Milstein A Tool detection and operative skill assessment in surgical videos using region-based convolutional neural networks. In: 2018 IEEE Winter Conference on Applications of Computer Vision (WACV) (2018a). p. 691–9. 10.1109/WACV.2018.00081

[62] Zhang B Fung A Torabi M Barker J Foley G Abukhalil R C-ECT: online surgical phase recognition with cross-enhancement causal transformer. In: 2023 IEEE 20th International Symposium on Biomedical Imaging (ISBI), (Cartagena, Colombia: IEEE) (2023a). p. 1–5. 10.1109/ISBI53787.2023.10230841

[63] Zheng M Ye M Rafii-Tari H. Automatic biopsy tool presence and episode recognition in robotic bronchoscopy using a multi-task vision transformer network. In: 2022 International Conference on Robotics and Automation (ICRA); 7349–55; Philadelphia, PA, USA: IEEE (2022). 10.1109/ICRA46639.2022.9811982

[64] Kim JW Zhang P Gehlbach P Iordachita I Kobilarov M. Towards autonomous eye surgery by combining deep imitation learning with optimal control. Proc Mach Learn Res*.* (2021) 155:2347–58. 34712957 PMC8549631

[65] Abdulbaki Alshirbaji T Aldeen Jalal N Docherty PD Neumuth T Moeller K. A comparative evaluation of spatial pooling methods for surgical tool detection. Curr Dir Biomed Eng. (2023) 9:214–7. 10.1515/cdbme-2023-1054

[66] Arabian H Abdulbaki Alshirbaji T Jalal NA Krueger-Ziolek S Moeller K. P-CSEM: an attention module for improved laparoscopic surgical tool detection. Sensors. (2023) 23:7257. 10.3390/s23167257 37631791 PMC10459566

[67] Reiter W. Domain generalization improves end-to-end object detection for real-time surgical tool detection. Int J Comput Assist Radiol Surg. (2022) 18:939–44. 10.1007/s11548-022-02823-9 36581742

[68] Jalal NA Alshirbaji TA Docherty PD Arabian H Laufer B Krueger-Ziolek S Laparoscopic video analysis using temporal, attention, and multi-feature fusion based-approaches. Sensors. (2023) 23:1958. 10.3390/s23041958 36850554 PMC9964851

[69] Yamlahi A Tran TN Godau P Schellenberg M Michael D Smidt F-H Self-distillation for surgical action recognition. In: Greenspan H Madabhushi A Mousavi P Salcudean S Duncan J Syeda-Mahmood T editors. Medical Image Computing and Computer Assisted Intervention – MICCAI 2023. Cham: Springer Nature Switzerland (2023). p. 637–46. 10.1007/978-3-031-43996-4_61

[70] Arabian H Dalla FA Jalal NA Alshirbaji TA Moeller K. Attention networks for improving surgical tool classification in laparoscopic videos. Curr Dir Biomed Eng. (2022) 8:676–9. 10.1515/cdbme-2022-1172

[71] Abdulbaki Alshirbaji T Aldeen Jalal N Docherty PD Neumuth T Moeller K. Neural network classification of surgical tools in gynecological videos. Curr Dir Biomed Eng*.* (2022) 8:644–7. 10.1515/cdbme-2022-1164

[72] Philipp M Alperovich A Gutt-Will M Mathis A Saur S Raabe A Dynamic CNNs using uncertainty to overcome domain generalization for surgical instrument localization. In: 2022 IEEE/CVF Winter Conference on Applications of Computer Vision (WACV). Waikoloa, HI, USA: IEEE (2022). p. 1727–36. 10.1109/WACV51458.2022.00179

[73] Aspart F Bolmgren JL Lavanchy JL Beldi G Woods MS Padoy N Clipassistnet: bringing real-time safety feedback to operating rooms. Int J CARS. (2022) 17:5–13. 10.1007/s11548-021-02441-x PMC8739308 34297269

[74] Alshirbaji TA Jalal NA Docherty PD Neumuth T Moeller K. Assessing generalisation capabilities of CNN models for surgical tool classification. Curr Dir Biomed Eng*.* (2021) 7:476–9. 10.1515/cdbme-2021-2121

[75] Abdulbaki Alshirbaji T Jalal NA Docherty PD Neumuth T Möller K. A deep learning spatial-temporal framework for detecting surgical tools in laparoscopic videos. Biomed Signal Process Control. (2021) 68:102801. 10.1016/j.bspc.2021.102801

[76] Jalal NA Alshirbaji TA Docherty PD Neumuth T Moeller K. A deep learning framework for recognising surgical phases in laparoscopic videos. IFAC Pap. (2021) 54:334–9. 10.1016/j.ifacol.2021.10.278

[77] Jalal NA Abdulbaki Alshirbaji T Docherty PD Neumuth T Moeller K. Surgical tool detection in laparoscopic videos by modeling temporal dependencies between adjacent frames. In: Jarm T Cvetkoska A Mahnič-Kalamiza S Miklavcic D, editors. 8th European Medical and Biological Engineering Conference. Cham: Springer International Publishing (2021). p. 1045–52. 10.1007/978-3-030-64610-3_117

[78] Bieck R Heuermann K Pirlich M Neumann J Neumuth T. Language-based translation and prediction of surgical navigation steps for endoscopic wayfinding assistance in minimally invasive surgery. Int J Comput Assist Radiol Surg*.* (2020) 15:2089–100. 10.1007/s11548-020-02264-2 33037490 PMC7671992

[79] Ivantsits M Tautz L Sündermann S Wamala I Kempfert J Kuehne T DL-based segmentation of endoscopic scenes for mitral valve repair. Curr Dir Biomed Eng. (2020) 6:20200017. 10.1515/cdbme-2020-0017

[80] Tamer Abdulbaki A Jalal NA Möller K. A convolutional neural network with a two-stage LSTM model for tool presence detection in laparoscopic videos. Curr Dir Biomed Eng*.* (2020) 6:20200002. 10.1515/cdbme-2020-0002

[81] Sahu M Szengel A Mukhopadhyay A Zachow S. Surgical phase recognition by learning phase transitions. Curr Dir Biomed Eng. (2020) 6:20200037. 10.1515/cdbme-2020-0037

[82] Jalal NA Alshirbaji TA Möller K. Predicting surgical phases using CNN-NARX neural network. Curr Dir Biomed Eng*.* (2019) 5:405–7. 10.1515/cdbme-2019-0102

[83] Bodenstedt S Active learning using deep Bayesian networks for surgical workflow analysis. Int J Comput Assist Radiol Surg*.* (2019) 14:1079–87. 10.1007/s11548-019-01963-9 30968355

[84] Funke I Bodenstedt S Oehme F Von Bechtolsheim F Weitz J Speidel S Using 3D convolutional neural networks to learn spatiotemporal features for automatic surgical gesture recognition in video. In: Shen D Liu T Peters TM Staib LH Essert C Zhou S, editors. Medical Image Computing and Computer Assisted Intervention – MICCAI 2019. Cham: Springer International Publishing (2019). p. 467–75. 10.1007/978-3-030-32254-0_52

[85] Prellberg J Kramer O. Multi-label classification of surgical tools with convolutional neural networks. In: 2018 International Joint Conference on Neural Networks (IJCNN); 1-8; Rio de Janeiro; IEEE; (2018). 10.1109/IJCNN.2018.8489647

[86] Abdulbaki Alshirbaji T Jalal NA Möller K. Surgical tool classification in laparoscopic videos using convolutional neural network. Curr Dir Biomed Eng. (2018) 4:407–10. 10.1515/cdbme-2018-0097

[87] Petscharnig S Schoffmann K Benois-Pineau J Chaabouni S Keckstein J. Early and late fusion of temporal information for classification of surgical actions in laparoscopic gynecology. In: 2018 IEEE 31st International Symposium on Computer-Based Medical Systems (CBMS); 369–74; Karlstad; IEEE; (2018). 10.1109/CBMS.2018.00071

[88] Nwoye CI Yu T Sharma S Murali A Alapatt D Vardazaryan A Cholectriplet2022: show me a tool and tell me the triplet — an endoscopic vision challenge for surgical action triplet detection. Med Image Anal. (2023b) 89:102888. 10.1016/j.media.2023.102888 37451133

[89] Ramesh S Srivastav V Alapatt D Yu T Murali A Sestini L Dissecting self-supervised learning methods for surgical computer vision. Med Image Anal. (2023) 88:102844. 10.1016/j.media.2023.102844 37270898

[90] Sharma S Nwoye CI Mutter D Padoy N. Rendezvous in time: an attention-based temporal fusion approach for surgical triplet recognition. Int J Comput Assist Radiol Surg*.* (2023) 18:1053–9. 10.1007/s11548-023-02914-1 37097518

[91] Nwoye CI Alapatt D Yu T Vardazaryan A Xia F Zhao Z Cholectriplet2021: a benchmark challenge for surgical action triplet recognition. Med Image Anal. (2023a) 86:102803. 10.1016/j.media.2023.102803 37004378

[92] Madad Zadeh S François T Comptour A Canis M Bourdel N Bartoli A. SurgAI3.8K: a labeled dataset of gynecologic organs in laparoscopy with application to automatic augmented reality surgical guidance. J Minim Invasive Gynecol. (2023) 30:397–405. 10.1016/j.jmig.2023.01.012 36720429

[93] Sestini L Rosa B De Momi E Ferrigno G Padoy N. FUN-SIS: a fully unsupervised approach for surgical instrument segmentation. Med Image Anal*.* (2023) 85:102751. 10.1016/j.media.2023.102751 36716700

[94] Takeuchi M Collins T Ndagijimana A Kawakubo H Kitagawa Y Marescaux J Automatic surgical phase recognition in laparoscopic inguinal hernia repair with artificial intelligence. Hernia. (2022) 26:1669–78. 10.1007/s10029-022-02621-x 35536371

[95] Mascagni P Alapatt D Laracca GG Guerriero L Spota A Fiorillo C Multicentric validation of EndoDigest: a computer vision platform for video documentation of the critical view of safety in laparoscopic cholecystectomy. Surg Endosc. (2022a) 36:8379–86. 10.1007/s00464-022-09112-1 35171336

[96] Nwoye CI Yu T Gonzalez C Seeliger B Mascagni P Mutter D Rendezvous: attention mechanisms for the recognition of surgical action triplets in endoscopic videos. Med Image Anal. (2022) 78:102433. 10.1016/j.media.2022.102433 35398658

[97] Huaulmé A Sarikaya D Le Mut K Despinoy F Long Y Dou Q MIcro-surgical anastomose workflow recognition challenge report. Comput Methods Programs Biomed. (2021) 212:106452. 10.1016/j.cmpb.2021.106452 34688174

[98] Ramesh S Dall'Alba D Gonzalez C Yu T Mascagni P Mutter D Multi-task temporal convolutional networks for joint recognition of surgical phases and steps in gastric bypass procedures. Int J CARS. (2021) 16:1111–9. 10.1007/s11548-021-02388-z PMC8260406 34013464

[99] Nwoye CI Gonzalez C Yu T Mascagni P Mutter D Marescaux J Recognition of instrument-tissue interactions in endoscopic videos via action triplets. In: Martel AL Abolmaesumi P Stoyanov D Mateus D Zuluaga MA Zhou SK, editors. Medical Image Computing and Computer Assisted Intervention – MICCAI 2020. Cham: Springer International Publishing (2020). 364–74. 10.1007/978-3-030-59716-0_35

[100] Rosa B Bordoux V Nageotte F. Combining differential kinematics and optical flow for automatic labeling of Continuum robots in minimally invasive surgery. Front Robot AI. (2019) 6:86. 10.3389/frobt.2019.00086 33501101 PMC7805658

[101] Nwoye CI Mutter D Marescaux J Padoy N. Weakly supervised convolutional LSTM approach for tool tracking in laparoscopic videos. Int J Comput Assist Radiol Surg. (2019) 14:1059–67. 10.1007/s11548-019-01958-6 30968356

[102] Al Hajj H Lamard M Conze P-H Roychowdhury S Hu X Maršalkaitė G CATARACTS: challenge on automatic tool annotation for CATARACT surgery. Med Image Anal*.* (2019) 52:24–41. 10.1016/j.media.2018.11.008 30468970

[103] Vardazaryan A Mutter D Marescaux J Padoy N. Weakly- Supervised learning for tool localization in laparoscopic videos. In: Stoyanov D Taylor Z Balocco S Sznitman R Martel A Maier-Hein L, editors. Intravascular Imaging and Computer Assisted Stenting and Large-Scale Annotation of Biomedical Data and Expert Label Synthesis. Cham: Springer International Publishing (2018). p. 169–79. 10.1007/978-3-030-01364-6_19

[104] Huber M Ourselin S Bergeles C Vercauteren T. Deep homography prediction for endoscopic camera motion imitation learning. In Greenspan H Madabhushi A Mousavi P Salcudean S Duncan J Syeda-Mahmood T, editors. Medical Image Computing and Computer Assisted Intervention – MICCAI 2023. Cham: Springer Nature Switzerland (2023). p. 217–26. 10.1007/978-3-031-43996-4_21

[105] Lam K Lo FP-W An Y Darzi A Kinross JM Purkayastha S Deep learning for instrument detection and assessment of operative skill in surgical videos. IEEE Trans Med Robot Bionics. (2022) 4:1068–71. 10.1109/TMRB.2022.3214377

[106] Jin Y Yu Y Chen C Zhao Z Heng P-A Stoyanov D. Exploring intra- and inter-video relation for surgical semantic scene segmentation. IEEE Trans Med Imaging. (2022) 41:2991–3002. 10.1109/TMI.2022.3177077 35604967

[107] Gupta S Ali S Goldsmith L Turney B Rittscher J. Multi-class motion-based semantic segmentation for ureteroscopy and laser lithotripsy. Comput Med Imaging Graph*.* (2022) 101:102112. 10.1016/j.compmedimag.2022.102112 36030620

[108] Tukra S Marcus HJ Giannarou S. See-through vision with unsupervised scene occlusion reconstruction. IEEE Trans Pattern Anal Mach Intell*.* (2022) 1:10.1109/TPAMI.2021.3058410. 10.1109/TPAMI.2021.3058410 33566758

[109] Kadkhodamohammadi A Luengo I Stoyanov D. PATG: position-aware temporal graph networks for surgical phase recognition on laparoscopic videos. Int J Comput Assist Radiol Surg*.* (2022) 17:849–56. 10.1007/s11548-022-02600-8 35353299

[110] Zhang Y Bano S Page A-S Deprest J Stoyanov D Vasconcelos F. Large-scale surgical workflow segmentation for laparoscopic sacrocolpopexy. Int J CARS. (2022b) 17:467–77. 10.1007/s11548-021-02544-5 PMC8873061 35050468

[111] Zhang FX Al Moubayed N Shum HPH. Towards graph representation learning based surgical workflow anticipation. In: 2022 IEEE-EMBS International Conference on Biomedical and Health Informatics (BHI); 01–4; Ioannina, Greece; IEEE; (2022). 10.1109/BHI56158.2022.9926801

[112] Shao M Clark J Huson D Hardeberg J. Real-time style transfer for videos to enhance the realism of simulation of laparoscopic surgeries. In: 2022 10th European Workshop on Visual Information Processing (EUVIP); 1–6; IEEE; Lisbon, Portugal; (2022). 10.1109/EUVIP53989.2022.9922706

[113] Grammatikopoulou M Flouty E Kadkhodamohammadi A Quellec G Chow A Nehme J CaDIS: cataract dataset for surgical RGB-image segmentation. Med Image Anal. (2021) 71:102053. 10.1016/j.media.2021.102053 33864969

[114] Garcia-Peraza-Herrera LC Fidon L D’Ettorre C Stoyanov D Vercauteren T Ourselin S. Image compositing for segmentation of surgical tools without manual annotations. IEEE Trans Med Imaging. (2021) 40:1450–60. 10.1109/TMI.2021.3057884 33556005 PMC8092331

[115] Sharma C Singh H Orihuela-Espina F Darzi A Sodergren MH. Visual gaze patterns reveal surgeons’ ability to identify risk of bile duct injury during laparoscopic cholecystectomy. HPB (Oxford). (2021) 23:715–22. 10.1016/j.hpb.2020.09.007 32988756

[116] Colleoni E Stoyanov D. Robotic instrument segmentation with image-to-image translation. IEEE Robot Autom Lett*.* (2021) 6:935–42. 10.1109/LRA.2021.3056354

[117] Ravasio CS Pissas T Bloch E Flores B Jalali S Stoyanov D Learned optical flow for intra-operative tracking of the retinal fundus. Int J CARS. (2020) 15:827–36. 10.1007/s11548-020-02160-9 PMC7261285 32323210

[118] Colleoni E Edwards P Stoyanov D Synthetic and real inputs for tool segmentation in robotic surgery. In: Martel AL Abolmaesumi P Stoyanov D Mateus D Zuluaga MA Zhou SK, editors. Medical Image Computing and Computer Assisted Intervention – MICCAI 2020. Cham: Springer International Publishing (2020). p. 700–10. 10.1007/978-3-030-59716-0_67

[119] Fuentes-Hurtado F Kadkhodamohammadi A Flouty E Barbarisi S Luengo I Stoyanov D. Easylabels: weak labels for scene segmentation in laparoscopic videos. Int J CARS. (2019) 14:1247–57. 10.1007/s11548-019-02003-2 31165349

[120] Du X Kurmann T Chang P-L Allan M Ourselin S Sznitman R Articulated multi-instrument 2-D pose estimation using fully convolutional networks. IEEE Trans Med Imaging. (2018) 37:1276–87. 10.1109/TMI.2017.2787672 29727290 PMC6051486

[121] Zisimopoulos O Flouty E Luengo I Giataganas P Nehme J Chow A Deepphase: surgical phase recognition in CATARACTS videos. In: Frangi AF Schnabel JA Davatzikos C Alberola-López C Fichtinger G, editors. Medical Image Computing and Computer Assisted Intervention – MICCAI 2018. Cham: Springer International Publishing (2018). p. 265–72. 10.1007/978-3-030-00937-3_31

[122] Mascagni P Alapatt D Sestini L Altieri MS Madani A Watanabe Y Computer vision in surgery: from potential to clinical value. Npj digit. Med. (2022b) 5:163. 10.1038/s41746-022-00707-5 PMC9616906 36307544

[123] Colleoni E Moccia S Du X De Momi E Stoyanov D. Deep learning based robotic tool detection and articulation estimation with spatio-temporal layers. IEEE Robot Autom Lett*.* (2019) 4:2714–21. 10.1109/LRA.2019.2917163

[124] Shi X Jin Y Dou Q Heng P-A. Semi-supervised learning with progressive unlabeled data excavation for label-efficient surgical workflow recognition. Med Image Anal*.* (2021) 73:102158. 10.1016/j.media.2021.102158 34325149

[125] Jin Y Long Y Chen C Zhao Z Dou Q Heng P-A. Temporal memory relation network for workflow recognition from surgical video. IEEE Trans Med Imaging. (2021) 40:1911–23. 10.1109/TMI.2021.3069471 33780335

[126] Shi X Jin Y Dou Q Heng P-A. LRTD: long-range temporal dependency based active learning for surgical workflow recognition. Int J Comput Assist Radiol Surg*.* (2020) 15:1573–84. 10.1007/s11548-020-02198-9 32588246

[127] Jin Y Li H Dou Q Chen H Qin J Fu C-W Multi-task recurrent convolutional network with correlation loss for surgical video analysis. Med Image Anal. (2020) 59:101572. 10.1016/j.media.2019.101572 31639622

[128] Zheng Q Yang R Yang S Ni X Li Y Jiang Z Development and validation of a deep-learning based assistance system for enhancing laparoscopic control level. Rob Comput Surg. (2023) 19:e2449. 10.1002/rcs.2449 35922092

[129] Zou X Liu W Wang J Tao R Zheng G. ARST: auto-regressive surgical transformer for phase recognition from laparoscopic videos. Comput Methods Biomech Biomed Eng Imaging Vis*.* (2023) 11:1012–8. 10.1080/21681163.2022.2145238

[130] Jin Y Cheng K Dou Q Heng P-A Incorporating temporal prior from motion flow for instrument segmentation in minimally invasive surgery video. In: Shen D Liu T Peters TM Staib LH Essert C Zhou S, editors. Medical Image Computing and Computer Assisted Intervention – MICCAI 2019. Cham: Springer International Publishing (2019). p. 440–8. 10.1007/978-3-030-32254-0_49

[131] Zhang Y Kim M Jin S. Real-time detection and tracking of surgical instrument based on YOLOv5 and DeepSORT*. In: 2023 32nd IEEE International Conference on Robot and Human Interactive Communication (RO-MAN); 1758–63; IEEE; Busan, Korea, Republic of; (2023). 10.1109/RO-MAN57019.2023.10309495

[132] Wang H Jin Y Zhu L. Dynamic interactive relation capturing via scene graph learning for robotic surgical report generation. In: 2023 IEEE International Conference on Robotics and Automation (ICRA); 2702–9; IEEE; London, United Kingdom; (2023). 10.1109/ICRA48891.2023.10160647

[133] Kitaguchi D Fujino T Takeshita N Hasegawa H Mori K Ito M. Limited generalizability of single deep neural network for surgical instrument segmentation in different surgical environments. Sci Rep. (2022) 12:12575. 10.1038/s41598-022-16923-8 35869249 PMC9307578

[134] Ata YM Al-Jassim FA Alabassi K Albakr A Ismail T Al Jalham K. Pelvic lipomatosis—a rare diagnosis and a challenging management: a case report and literature review. J Surg Case Rep. (2024) 2024:rjae777. 10.1093/jscr/rjae777 39678478 PMC11646686

[135] Khalil IA Alani A Al Saeedi A Shehadeh H Ahmed T Al-Jalham K. Life-threatening abdominal compartment syndrome as a complication of supine super mini percutaneous nephrolithotomy, the first case report and literature review. Urol Case Rep. (2021) 36:101578. 10.1016/j.eucr.2021.101578 33537210 PMC7840850

[136] Wang F Sun X Li J. Surgical smoke removal via residual swin transformer network. Int J Comput Assist Radiol Surg*.* (2023) 18:1417–27. 10.1007/s11548-023-02835-z 36683136

[137] Hayoz M Hahne C Gallardo M Candinas D Kurmann T Allan M Learning how to robustly estimate camera pose in endoscopic videos. Int J CARS. (2023) 18:1185–92. 10.1007/s11548-023-02919-w PMC10329609 37184768

[138] Doughty M Ghugre NR. HMD-EgoPose: head-mounted display-based egocentric marker-less tool and hand pose estimation for augmented surgical guidance. Int J Comput Assist Radiol Surg. (2022) 17:2253–62. 10.1007/s11548-022-02688-y 35701681

[139] Bamba Y Ogawa S Itabashi M Shindo H Kameoka S Okamoto T Object and anatomical feature recognition in surgical video images based on a convolutional neural network. Int J CARS. (2021) 16:2045–54. 10.1007/s11548-021-02434-w PMC8224261 34169465

[140] Tanzi L Piazzolla P Porpiglia F Vezzetti E. Real-time deep learning semantic segmentation during intra-operative surgery for 3D augmented reality assistance. Int J Comput Assist Radiol Surg. (2021) 16:1435–45. 10.1007/s11548-021-02432-y 34165672 PMC8354939

[141] Loukas C Frountzas M Schizas D. Patch-based classification of gallbladder wall vascularity from laparoscopic images using deep learning. Int J Comput Assist Radiol Surg. (2021) 16:103–13. 10.1007/s11548-020-02285-x 33146850

[142] Zhang J Gao X. Object extraction via deep learning-based marker-free tracking framework of surgical instruments for laparoscope-holder robots. Int J Comput Assist Radiol Surg. (2020) 15:1335–45. 10.1007/s11548-020-02214-y 32577985

[143] Nakazawa A Harada K Mitsuishi M Jannin P. Real-time surgical needle detection using region-based convolutional neural networks. Int J Comput Assist Radiol Surg. (2020) 15:41–7. 10.1007/s11548-019-02050-9 31422553

[144] Bhattarai A Alsadoon A Prasad PWC Pham L Haddad S Hsu J A novel multiple communication paths for surgical telepresence videos delivery of the maxilla area in oral and maxillofacial surgery. Int J CARS. (2019) 14:873–83. 10.1007/s11548-018-01904-y 30649669

[145] Gupta G Shankar S Pinisetty S. Automated surgical procedure assistance framework using deep learning and formal runtime monitoring. In: Dang T Stolz V, editors. Runtime Verification. Cham: Springer International Publishing (2022). 25–44. vol. 13498.

[146] Valderrama N Ruiz Puentes P Hernández I Ayobi N Verlyck M Santander J Towards holistic surgical scene understanding. In: Wang L Dou Q Fletcher PT Speidel S Li S, editors. Medical Image Computing and Computer Assisted Intervention – MICCAI 2022. Cham: Springer Nature Switzerland (2022). p. 442–52. 10.1007/978-3-031-16449-1_42

[147] Schmidt A Mohareri O DiMaio S Salcudean SE. Recurrent implicit neural graph for deformable tracking in endoscopic videos. In: Wang L Dou Q Fletcher PT Speidel S Li S, editors. Medical Image Computing and Computer Assisted Intervention—MICCAI 2022. Cham: Springer Nature Switzerland (2022). 478–88. vol. 13434.

[148] Fathollahi M Sarhan MH Pena R DiMonte L Gupta A Ataliwala A Video-Based surgical skills assessment using long term tool tracking. In: Wang L Dou Q Fletcher PT Speidel S Li S, editors. Medical Image Computing and Computer Assisted Intervention – MICCAI 2022. Cham: Springer Nature Switzerland (2022). p. 541–50. 10.1007/978-3-031-16449-1_52

[149] Kalia M Aleef TA Navab N Black P Salcudean SE Co-generation and segmentation for generalized surgical instrument segmentation on unlabelled data. In: De Bruijne M Cattin PC Cotin S Padoy N Speidel S Zheng Y, editors. Medical Image Computing and Computer Assisted Intervention – MICCAI 2021. Cham: Springer International Publishing (2021). p. 403–12. 10.1007/978-3-030-87202-1_39

[150] Yang Y Zhao Z Shi P Hu S. An efficient one-stage detector for real-time surgical tools detection in robot-assisted surgery. In: Papież BW Yaqub M Jiao J Namburete AIL Noble JA, editors. Medical Image Understanding and Analysis. Cham: Springer International Publishing (2021) 18–29. vol. 12722.

[151] Hachiuma R Shimizu T Saito H Kajita H Takatsume Y. Deep selection: a fully supervised camera selection network for surgery recordings. In: Martel AL Abolmaesumi P Stoyanov D Mateus D Zuluaga MA Zhou SK, editors. Medical Image Computing and Computer Assisted Intervention—MICCAI 2020. Cham: Springer International Publishing (2020). 419–28. vol. 12263.

[152] Sokolova N Schoeffmann K Taschwer M Putzgruber-Adamitsch D El-Shabrawi Y Evaluating the generalization performance of instrument classification in cataract surgery videos. In: Ro YM Cheng W-H Kim J Chu W-T Cui P Choi J-W, editors. MultiMedia Modeling. Cham: Springer International Publishing (2020). p. 626–36. 10.1007/978-3-030-37734-2_51

[153] Primus MJ Putzgruber-Adamitsch D Taschwer M Münzer B El-Shabrawi Y Böszörmenyi L Frame-Based classification of operation phases in cataract surgery videos. In: Schoeffmann K Chalidabhongse TH Ngo CW Aramvith S O'Connor NE Ho Y-S, editors. MultiMedia Modeling. Cham: Springer International Publishing (2018). p. 241–53. 10.1007/978-3-319-73603-7_20

[154] Hasan Md K Calvet L Rabbani N, Bartoli A, Detection, segmentation, and 3D pose estimation of surgical tools using convolutional neural networks and algebraic geometry. Med Image Anal*.* (2021) 70:101994. 10.1016/j.media.2021.101994 33611053

[155] Feng X Zhang X Shi X Li L Wang S. ST-ITEF: spatio-temporal intraoperative task estimating framework to recognize surgical phase and predict instrument path based on multi-object tracking in keratoplasty. Med Image Anal*.* (2024) 91:103026. 10.1016/j.media.2023.103026 37976868

[156] Ping L Wang Z Yao J Gao J Yang S Li J Application and evaluation of surgical tool and tool tip recognition based on convolutional neural network in multiple endoscopic surgical scenarios. Surg Endosc. (2023) 37:7376–84. 10.1007/s00464-023-10323-3 37580576

[157] De Backer P Eckhoff JA Simoens J Müller DT Allaeys C Creemers H Multicentric exploration of tool annotation in robotic surgery: lessons learned when starting a surgical artificial intelligence project. Surg Endosc. (2022) 36:8533–48. 10.1007/s00464-022-09487-1 35941310

[158] Guédon ACP Meij SEP Osman KNMMH Kloosterman HA Van Stralen KJ Grimbergen MCM Deep learning for surgical phase recognition using endoscopic videos. Surg Endosc. (2021) 35:6150–7. 10.1007/s00464-020-08110-5 33237461

[159] Tokuyasu T Iwashita Y Matsunobu Y Kamiyama T Ishikake M Sakaguchi S Development of an artificial intelligence system using deep learning to indicate anatomical landmarks during laparoscopic cholecystectomy. Surg Endosc. (2021) 35:1651–8. 10.1007/s00464-020-07548-x 32306111 PMC7940266

[160] Bian G-B Zhang L Chen H Li Z Fu P Yue W-Q Motion decoupling network for intra-operative motion estimation under occlusion. IEEE Trans Med Imaging. (2023) 42:2924–35. 10.1109/TMI.2023.3268774 37079409

[161] Yue W Liao H Xia Y Lam V Luo J Wang Z. Cascade multi-level transformer network for surgical workflow analysis. IEEE Trans Med Imaging. (2023) 42:2817–31. 10.1109/TMI.2023.3265354 37037257

[162] Padoy N. Machine and deep learning for workflow recognition during surgery. Minim Invasive Ther Allied Technol. (2019) 28:82–90. 10.1080/13645706.2019.1584116 30849261

[163] Colleoni E Moccia S Du X Stoyanov D. Deep Learning Based Robotic Tool Detection and Articulation Estimation with Spatio-Temporal Layers. Piscataway, NJ: IEEE Journals & Magazine|IEEE Xplore. (2019). 10.1109/LRA.2019.2917163

[164] Kitaguchi D Takeshita N Matsuzaki H Oda T Watanabe M Mori K Automated laparoscopic colorectal surgery workflow recognition using artificial intelligence: experimental research. Int J Surg. (2020) 79:88–94. 10.1016/j.ijsu.2020.05.015 32413503

[165] Yu F Silva Croso G Kim TS Song Z Parker F Hager GD Assessment of automated identification of phases in videos of cataract surgery using machine learning and deep learning techniques. JAMA Netw Open. (2019) 2:e191860. 10.1001/jamanetworkopen.2019.1860 PMC6450320 30951163

[166] Novel applications of deep learning in surgical training. In: De Pablos PO Zhang X, editors. Artificial Intelligence, Big Data, Blockchain and 5G for the Digital Transformation of the Healthcare Industry. Amsterdam: Academic Press (2024). 301–20. 10.1016/B978-0-443-21598-8.00021-X

[167] Wang Y Wu Z Morgan TN Garbens A. Evaluating robotic-assisted partial nephrectomy surgeons with fully convolutional segmentation and multi-task attention networks. J Robot Surg. (2023) 17:2323–30. 10.1007/s11701-023-01657-0 37368225 PMC10492672

[168] Ahmed FA Arsalan M Al-Ali A Al-Jalham K Balakrishnan S. CLIP-RL: surgical scene segmentation using contrastive language-vision pretraining & reinforcement learning. arXiv [Preprint]. arXiv:2507.04317 (2025). 10.48550/ARXIV.2507.04317

[169] Abdel-Ghani M Ali M Ali M Ahmed F Arsalan M Al-Ali A FASL-Seg: anatomy and tool segmentation of surgical scenes. In: Lynce I Murano N Vallati M Villata S Chesani F Milano M editors. Frontiers in Artificial Intelligence and Applications. Amsterdam: IOS Press (2025). 10.3233/FAIA250908

[170] Ahmed F Abdel-Ghani M Arsalan M Ali M Al-Ali A Balakrishnan S. Surg-SegFormer: a dual transformer-based model for holistic surgical scene segmentation. In: 2025 IEEE 21st International Conference on Automation Science and Engineering (CASE). Los Angeles, CA, USA: IEEE (2025). p. 1304–9. 10.1109/CASE58245.2025.11163962

[171] Kharbech S Abinahed J Aboumarzouk O Ansari WE Al Ansari A Balakrishnan S. Digital tools and innovative healthcare solutions: serious games and gamification in surgical training and patient care. In: De Pablos PO Zhang X, editors. Artificial Intelligence, Big Data, Blockchain and 5G for the Digital Transformation of the Healthcare Industry. Amsterdam: Academic Press (2024). 321–39. 10.1016/B978-0-443-21598-8.00007-5

[172] Shabir D Anbatawi M Padhan J Balakrishnan S Al-Ansari A Abinahed J Evaluation of user-interfaces for controlling movements of virtual minimally invasive surgical instruments. Robot Comput Surg. (2022) 18:e2414. 10.1002/rcs.2414 35486635

